# The heart rate method for estimating oxygen uptake: Analyses of reproducibility using a range of heart rates from cycle commuting

**DOI:** 10.1371/journal.pone.0219741

**Published:** 2019-07-24

**Authors:** Peter Schantz, Jane Salier Eriksson, Hans Rosdahl

**Affiliations:** The Research Unit for Movement, Health and Environment, The Åstrand Laboratory & Laboratory for Applied Sport Science, The Swedish School of Sport and Health Sciences, GIH, Stockholm, Sweden; University of Bourgogne France Comté, FRANCE

## Abstract

Monitoring aerobic exercise intensities of free-living physical activities is valuable for purposes such as education and research. The heart rate (HR) method, based on the linear relation between HR and oxygen uptake (VO_2_), is potentially valuable for this purpose. Three prerequisites are that the method is reproducible, and valid for the specific form of physical activity executed as well as under field conditions. The aim of this study is to evaluate reproducibility of the heart rate method in the laboratory. VO_2_ and HR measurements were made on two different occasions during three submaximal (model 1) plus a maximal exercise intensity (model 2) on a cycle ergometer in the laboratory. 19 habitual commuter cyclists (9 males and 10 females), aged 44 ± 3 years, were measured. The reproducibility of the estimated VO_2_, based on three levels of HR from commuting cycling and the regression equations from test and retest were analyzed. Differences between the two models were also studied. For both models, there were no significant differences between test and retest in the constituents of the regression equations (y-intercept, slope and r-value). Neither were there any systematic differences in estimated absolute levels of VO_2_ between test and retest. The relative differences between test and retest, based on estimations from three different levels of HR, were 0.99 ± 11.0 (n.s.), 2.67 ± 6.48 (n.s.) and 3.57 ± 6.24% (p<0.05) for model 1, and 1.09 ± 10.6, 1.75 ± 6.43 and 2.12 ± 5.92% (all n.s.) for model 2. However, some large individual differences were seen in both models. There were no significant differences between the two models in the slopes, intercepts or r-values of the regression equations or in the estimated levels of VO_2_. The heart rate method shows good reproducibility on the group level in estimating oxygen consumption from HR-VO_2_ relations in the laboratory, and based on three levels of HR which are representative for cycle commuting. However, on the individual level, some large variations were seen.

## Introduction

Monitoring metabolic demands and physiological work intensities of physical activities in field conditions is of great value in e.g. education and research. Portable instruments for indirect calorimetric measurements have been developed, but they are costly, technically complicated, and they can be sensitive to ambient conditions [1,2,3], which makes them difficult to use on a large scale. Furthermore, relevant methodological evaluations of them in laboratory [4] or in field conditions [2,3] are rare.

This motivates a renewed interest in the heart rate method (HR method). It is based on a linear relationship between heart rate (HR) and work rate/oxygen uptake (VO_2_) during exercise, as described early in the 20^th^ century [5,6,7,8]. Since then, HR recordings from various physical activities have been used in numerous studies as a basis for interpreting energy requirements and exercise intensities in humans [9,10,11,12,13] as well as in animals [14]. The value of such measurements is greater if individual HR-VO_2_ relations are established [11], which is facilitated by portable heart rate recorders and automatized stationary metabolic measurement devices. Furthermore, the relation between standardized work rates on ergometer cycles and VO_2_ can be used as a substitute for measuring VO_2_ [10,15,16], if taking into account that body weight affects the VO_2_ demands at standardized work loads [17,18,19]. Thus, the HR method can also be applied for purposes in which the exact levels of VO_2_ are not necessary to establish, such as in health education, promotion and surveillance.

However, the mentioned practice of using a method is one thing, validity and reproducibility is another. Already Berggren and Hohwü Christensen stated in 1950 [8] that the HR method must be used “with great care” since the HR “can vary independent of metabolic rate.” There are a number of issues related to validity of the HR method, e.g. the external validity of the HR-VO_2_ relations from laboratory to field settings, and to various types of physical activities with different durations and ambient conditions, that need to be studied in their own rights. Here we instead focus on the fundamental need of evaluating the reproducibility of the HR method under controlled laboratory conditions and to, in relation to previous studies, further the methodological approaches used.

Studies have indicated that the HR response to a repeated standarized cycle ergometer work rate may be erratic [11]. One reason for a non-stability is a habituation effect of varying magnitude, but leading to a lower pulse rate at a given submaximal work rate [20,21,22,23]. Another reason for instability in the HR response is a non-systematic day to day variability [8,11,12]. Whereas habituation effects can be handled through pre-test trials, a day to day variability is more difficult to circumvent, and can jeopardize the reproducibility of the HR-VO_2_ relation under both controlled laboratory and field conditions.

Pairs of HR and VO_2_ data, established at multiple submaximal and maximal work rates are normally used to calculate a linear regression equation for the HR method. It is thereby relevant to evaluate the reproducibility of it on the basis of the equations, as well as the outcomes of them, using different levels of HR to estimate VO_2_. Surprisingly enough, such evaluations have, to our knowledge, only been focussed on in two studies [24,25]. Both used a single HR level for their evaluations of the outcomes. One of the studies was dominated by patients with different clinical disorders [24]. Here the HR-VO_2_ relation was established from rest to low and intermediate work rates of walking and ergometer cycling. A great variability in the outcomes, based on rather low HR values from a 24 hour registration, led the authors to conclude that the “applied procedure seems unsuitable for metabolic studies in individual patients who engage in ordinary daily activities with low energy expenditure” [24]. McCrory et al. [25] studied the reproducibility in healthy subjects. Two different HR-VO_2_ relations were established based on measurements from resting to walking. Their single point HR evaluation was based on heart rate recordings from a normal day (ca. 15 hours). In the HR-VO_2_ relation, which was based solely on walking, a good reproducibility was noted on the group level, whereas a certain variability was noted on the individual levels.

**Fig 1:**
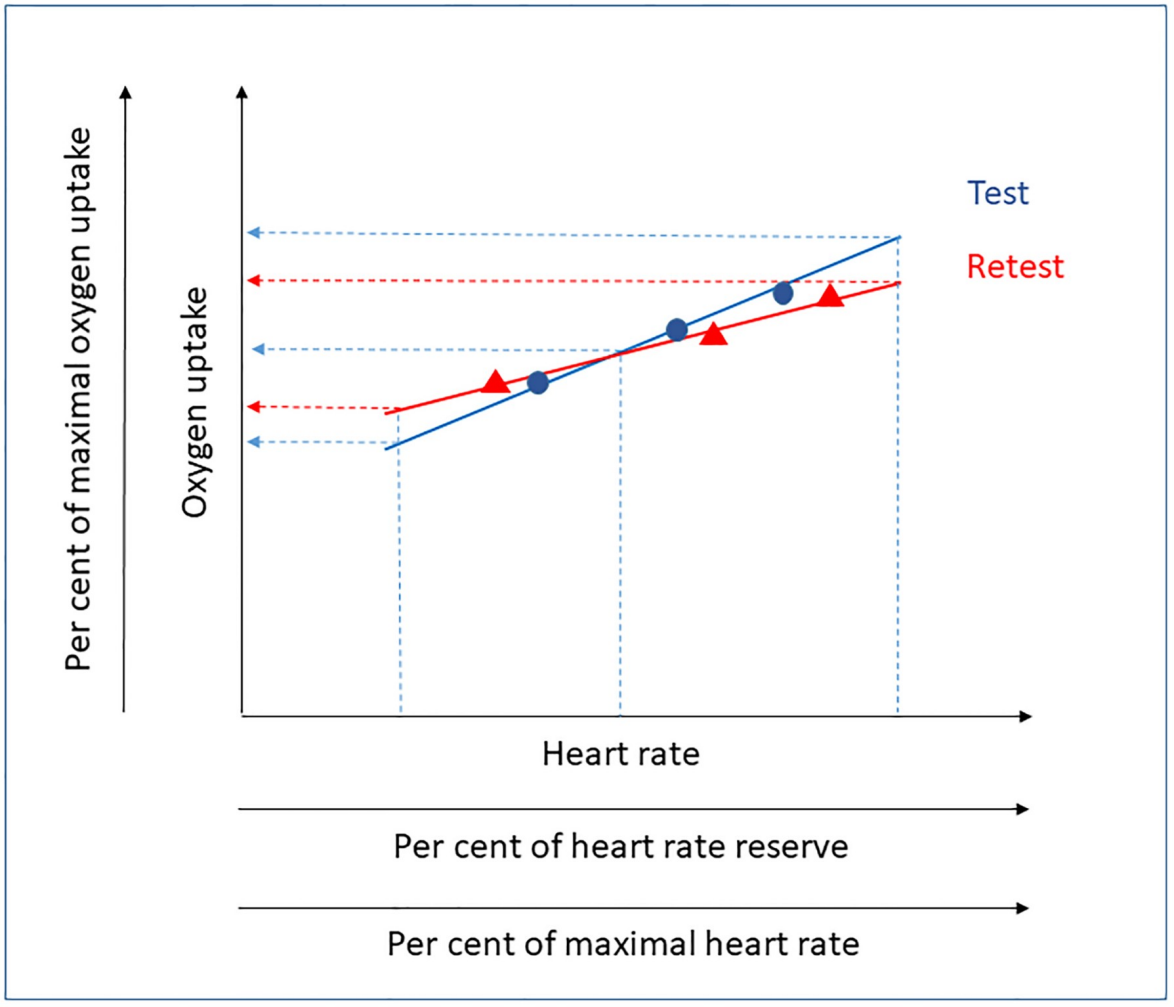
An illustration of how a variability in HR-VO_2_ relations at test and retest can affect measures of reproducibility. Linear relations from regression equations, based on values from three submaximal work rates at test and retest, are illustrated as unbroken lines. Based on different HR and the regression equations, the estimated levels of VO_2_ can be higher, equal or lower at test compared to retest (see broken lines).

The conflicting results, and evaluations based on only one, and rather low levels of HR, prompted us to further scrutinize these matters. Methodological issues being addressed relate to the degree of reproducibility possibly varying within one and the same study depending on the levels of HR used for the evaluations (Fig 1). If, for example, regression equation slopes from test and retest cross each other, an excellent reproducibility will be attained at the cross-point. However, on both sides of it, the absolute differences in estimated VO_2_ will increase, but in different directions. A great number of other potential interrelations between dual regression slopes and y-intercepts can produce a substantial variation in the test-retest variability. The magnitude of those differences may, however, be unimportant if they occur outside the relevant HR range. Thus, the reproducibility of VO_2_ estimations, based on HR-VO_2_ relations, needs to be studied at several HR levels that are distributed along a relevant range of HR.

Another factor, likely to determine the degree of reproducibility, is the number and span of work rates that are used to establish the HR-VO_2_ relations. To enable systematic studies of these matters it is therefore important to specify the HR levels used in terms of both absolute levels and percentages of maximal HR [26] as well as the heart rate reserve (HRR) [27,28]. The corresponding levels of VO_2_ and their percentage of the maximal oxygen uptake are also valuable to state (Fig 1). To our knowledge this has not been done before.

Given this background, the aim of the study was to evaluate day-to-day reproducibility of HR-VO_2_ regression equations (y-intercept, slope and correlation coefficient) and the estimated oxygen uptakes based on three levels of heart rates representative for everyday cycle commuting. Two HR-VO_2_ relations were established and compared, one with three levels of submaximal exercise (model 1), and another which also included a maximal exercise (model 2). The HR-VO_2_ relations were attained on an ergometer cycle in the laboratory for healthy and physically active middle-aged male and female cycle commuters.

## Methods

### Participants

Approval to conduct the study was obtained from the Ethics Committee North of the Karolinska Institute at the Karolinska Hospital (Dnr 03–637), Stockholm, Sweden.

#### Recruitment of participants

The process of selecting participants was divided into several steps. It started with advertisements in two major morning newspapers in Stockholm calling for participants. The inclusion criteria required being at least 20 years old; living in the County of Stockholm (excluding the municipality of Norrtälje), and walking or cycling the whole way, any distance, between home and place of work or study, and actively commuting in that fashion at least once a year. Answers could be sent in cost-free by post, fax, e-mail or by phone. These advertisements resulted in 2148 people volunteering to take part.

A questionnaire (The Physically Active Commuting in Greater Stockholm Questionnaire 1; PACS Q1, for it in Swedish and English, see Supporting information S1 and S2 Methods) was sent to these volunteers; 2010 were returned after three reminders. The questionnaire comprised 35 questions, but only the questions relevant for selecting our population were used in this study. These included gender, age, how physically strenuous their professional jobs were, commuting frequencies per week for each month of the year and commuting duration. The commuting distance of each individual was also used for selecting the study group. These were measured on routes drawn in maps by each respondent (For route mapping instructions, see Supporting information S3 and S4 Methods). The method for measuring the mapped distance is described in detail in Schantz and Stigell [29]. From the answers from PACS Q1, the respondents were divided into categories based on their reported mode of either cycling or walking, or combined modes.

Our sample was selected from the cyclist category, i.e. those subjects who only cycled to work. Other criteria were ages and route distances close to the median values of the male and female cyclists, respectively [30]. Candidates also rated their daily professional jobs as physically light or very light.

Information describing the physiological studies, test and standardization procedures as well as a health declaration was sent to the cyclists who fulfilled the criteria (for the information material, see Supporting information S5 and S6 Methods. The individual pictured in S5 and S6 Methods has provided written informed consent (as outlined in PLOS consent form) to publish his image alongside the manuscript. In the missive letter, the recipients were asked whether their previously drawn route was still valid, or of a comparable distance time-wise (comparable defined as plus/minus 5 to 10 minutes). If so, they were asked to respond to the health declaration which concerned whether they had any: 1) medication and kind of illness, 2) palpitations, chest pain or abnormally heavy breathing during exercise, 3) high blood pressure, or 4) had recently avoided or discontinued exercise for reasons of injury or health. The letter emphasized the right to terminate the tests at any time and without having to stipulate a reason. A signed informed consent of participation was returned.

Based on this information, individuals with invalid route distances and individuals with high blood pressure or medication that might affect normal heart rate were excluded. Anyone on medication with potential strong side-effects was also excluded. We contacted the remaining cyclists by telephone to settle potential questions, and to book test appointments. Telephone contacts continued until we had 9 men and 10 women who fulfilled the criteria and were willing to participate (Table 1). Based on the participants responses to a second questionnaire (PACS 2), that was sent to all responders of PACS 1 who wanted to participate in further studies, we could characterize all the participants in this study as non-smokers. The PACS 2 is presented as Supporting Information S7 and S8 Methods.

**Table 1:** Characteristics of the participants, their commuting cycle rides and environments (mean ± SD).

Cycle commuters	Age yrs	Height cm	Weight kg	BMI kg·m^-2^	Duration min	Distance km	Velocity km·h^-1^	Trips per year	Cycling environment*
**Males** (n = 9)	43 4	185 7	85 13	25 3	28.2 7.0	9.2 1.5	19.7 2.5	353 147	1.11 0.33
**Females** (n = 10)	44 3	170 5	66 7	23 3	23.2 5.0	6.5 1.5	16.8 1.9	348 110	1.10 0.74

Note:

* Cycling environment: 0 = inner urban; 1 = inner urban–suburban; 2 = suburban.

### Equipment and preparation

#### Stationary metabolic gas analysis system

A stationary metabolic gas analysis system (SMS), the Oxycon Pro (Carefusion GmbH, Hoechberg, Germany) was used in the mixing chamber mode for all metabolic measurements in the laboratory. The software used was JLAB 4.53. In this system the concentration of oxygen is measured with a paramagnetic analyser and the carbon dioxide concentration with an infra-red analyzer. The expired air is sampled continuously from the mixing chamber through a nafion tubing on the outside of the equipment connected to a nafion tubing on the inside of the equipment terminating at the analyzer inlets. Ventilation is measured through a digital volume transducer (DVT) which is attached to the outlet of the mixing chamber. The equipment was switched on 30 minutes before data collection and calibrated before and after each test using the built-in automated procedures and according to the manufacturer’s recommendations. The ambient conditions were first recorded, followed by calibration of the volume sensor and the gas analysers. A high precision gas of 15.00% O_2_, and 6.00% CO_2_ (accuracy: O_2_ ± 0.04% rel. and CO_2_ ± 0.1% rel. Air Liquid AB, Kungsängen, Sweden) was used for calibration.

A face mask with non-rebreathing air inlet valves (Combitox, Dräger Safety, Lübeck, Germany) was used. It was carefully fitted on the subject and checked for air leakage immediately prior to the measurements by the investigator and adjusted until no leakage occurred. For several subjects, a rubber insert was taped inside the top of the mask to prevent air leakage from the bridge of the nose. A tube (inner diameter of 35 mm) attached to the mask led the expired air into the mixing chamber. The measured variables were exported to Excel for further processing.

#### Ergometer cycle

A manually braked pendulum ergometer cycle (828E Monark Exercise AB, Vansbro, Sweden) was used. Before each experiment, the scale was zeroed while each subject sat on the saddle with his or her feet resting on the frame between the pedals, and hands resting on the handle bars. The saddle height was adjusted so that the participant’s knees were slightly flexed when the feet were on the pedals in their lowest position. The handle bars were adjusted to allow the participants to sit in an upright position. A digital metronome (DM70 Seiko S-Yard Co. Ltd, Tokyo, Japan) helped the subjects maintain the correct cadence while cycling. The work rate was controlled every minute by checking the cadence of the participant and the braking force as indicated on the pendulum scale.

#### Heart rate

HR was measured using a Polar Electro S610i Heart Rate Monitor, with a Polar Wearlink 31 transmitter (Polar Electro Oy, Kempele, Finland).

### Pre-measurement methodological studies

Prior to the data collection in the main study, an evaluation of two exercise protocols was undertaken to find the most suitable one for reaching maximal VO_2_ in normal healthy people. 14 other healthy participants (9 males and 5 females) were recruited among the staff and students at our workplace. The average age, height and weight for the males were 39.3 ± 11.9 years, 182.7 ± 9.0 cm and 82.6 ± 7.5 kg, and for the females 37.8 ± 11.1 years, 174.4 ± 6.8 cm and 65.4 ± 10.4 kg, respectively.

They cycled twice at three different submaximal work rates followed by an incremental maximal test. The order of the test protocols was randomized. These protocols are described in detail below under “Cycle ergometer exercise protocol”, the only difference being that the work rates during the maximal part were increased every 30 s or 60 s, respectively. A significant relative difference of 2.6 ± 4.2% in VO_2max_ was found between the two protocols (Table 2), favouring the use of the 60 s incremental protocol, which therefore was chosen for the maximal exercise tests in the main study.

**Table 2:** Comparison of test protocols for determining VO_2max_ and HR_max_ on a cycle ergometer (n = 14)(mean ± SD).

	Protocol A	Protocol B	Absolute differences	Relative differences, %	p-value abs. diff	p-value % diff
VO_2_ max, (L·min^-1^)	3.54 ± 0.68	3.45 ± 0.70	0.09 ± 0.16	2.60 ± 4.16	0.062	0.036
HR max, (beats·min^-1^)	188 ± 10	185 ± 8	2.64 ± 6.88	1.43 ± 3.72	0.174	0.174
Work rate max, (W)	290 ± 44	324 ± 48	-34 ± 15	-11 ± 4	0.000	0.000

Note: Increases in work rate: protocol A = every 60 s; protocol B = every 30 s.

### Measurements

#### Laboratory tests, standardization procedures and rest conditions

The participants’ responses were measured in the laboratory at rest, and submaximal as well as maximal work rates on two different occasions, which were completed within an average of 6.0 ± 7.3 days. Two trained investigators carried out the laboratory tests, each participant having the same investigator for each test. The participants were not able to drink during any of the tests.

The participants were asked to follow the same standard procedures before each test occasion. These were: 1) not to engage in any vigorous exercise for 24 hours beforehand, 2) not to cycle to the laboratory, 3) to refrain from eating, drinking, smoking and taking snuff (smokeless tobacco) for at least one hour before arrival at the laboratory, 4) not to eat a large meal at least three hours before the tests, 5) to avoid stress and 6) to cancel the test if they had fever, an infection or a cold. The time of day that the tests were undertaken was not standardised since it does not affect the HR-VO_2_ relation during physical activity [25]. The participants wore light clothes, such as T-shirts, shorts and training shoes, so as to diminish any effect of the energy liberation from the submaximal exercises on sweating and body temperature.

On arrival at the laboratory the participants were weighed and measured, and a check list was ticked off to determine whether they had followed the standard procedures named above. The participants then rested quietly for 10 minutes on a treatment table, and resting HR, in this case calculated from the time period between every single HR, was determined from the average of the five minutes between the 6^th^ and 10^th^ minutes.

#### Cycle ergometer exercise protocol

The participants cycled at three different work rates: 50, 100 and 150 watt (W) for the women, and 100, 150 and 200 W for the men. A cadence of 50 revolutions per minute (rpm) was chosen (p. 19 in [20]). At each work rate the participant cycled until steady-state (approximately 6 minutes), after which the resistance was increased. The third work rate was increased to only 125 W or 175 W for women and men respectively if, after the second work rate, the subject’s HR was higher than 150 beats per minute (bpm) and their perceived rate of exertion (RPE) according to a Borg scale exceeded 15 for both legs and breathing (p. 30 in [31]). The HR and the RPE were noted in the protocol after every minute.

Between the second and third work rates the test person continued cycling for 1 minute at a self-chosen low cadence with a resistance of 5 N. The subject was then instructed to resume the cadence of 50 rpm while the investigator slowly increased the work rate until, after one minute, the third work rate was reached. For that purpose, resistance was increased to 50 W during the first 15 seconds, to 100 W the second 15 seconds and successively to the required work rate during the last 30 seconds). Also, after the third submaximal test, the subject continued cycling for two minutes at a self-chosen low cadence at 5 N. For the submaximal tests in the laboratory, the mean of the four 15 s values for VO_2_ and HR for the last minute of each work rate were used for analysis.

During the maximal exercise phase, the subjects cycled at a cadence of 80 rpm [32]. For the first three minutes, the work rates were 60, 100, and 120 or 140 W for one minute each. The latter alternatives depended on which third work rate the subjects had during the submaximal work: 120 W if the third submaximal work rate had been 125 W or 175 W for women and men respectively; 140 W if it had been 150 W or 200 W for women and men, respectively. The work rate then increased by 20 W every 60 seconds. The test continued until exhaustion. HR was calculated for the whole minute before each increase of the resistance. The values for the maximal tests were calculated by averaging the highest four 15 s consecutive values for VO_2_ and HR at maximal exercise i.e. a collection period of 60 s [33]. The same corresponding values were used for both VO_2_ and HR.

To assess the RPE, a Borg scale was, as mentioned before, used [31]. The subjects were instructed on how to use the scale before commencing the tests. They were asked to point to a number on the scale that corresponded to their feeling of exertion for breathing and in their legs, respectively, before every increase of resistance during the submaximal test and directly after the maximal test. During the maximal phase they continued until exhaustion. To ensure that each subject achieved maximal exertion, at least two of the following three criteria were to be met by each subject: (i) a plateau in VO_2_ despite increasing exercise intensity (defined as a VO_2_ increment of less than 150 ml), (ii) a respiratory exchange ratio of ≥ 1.1, and (iii) a rating of RPE of ≥ 17 [33,34,35].

#### Measurements of heart rate and velocity during cycle commuting

The participants were met at their designated address by one of the investigators, who checked that the pre-test standardization procedures, as described above, had been followed. They commuted either to or from their work-place choosing themselves which time was most convenient. 17 of the cyclists (9 men; 8 women) were tested in the morning (start times between 06:58 h and 09:36 h) and the remaining two women were tested after work (start times 17:15 h and 17:37 h). The field trips took place in the inner urban and suburban–rural areas of Stockholm, Sweden. A detailed description of these areas and their boundaries can be found in Wahlgren and Schantz [36]. The majority of the participants (6 women and 7 men) cycle commuted from suburban to inner urban areas (cf. Table 1).

The participants were instructed to cycle at their normal pace, and their HR was measured continuously. They were not able to drink during their cycle commute. The mean values of the lowest, middle and highest fifth of heart rates during each participants´ cycle commuting were used to estimate the corresponding level of VO_2_ based on the HR-VO_2_ regression equations from the laboratory. These heart rate segments were determined through ordering all heart rates from the lowest to the highest, and then dividing them into segments of 1/5 of all heart rates. In each of these segments, the heart rates were normally distributed.

The starting time of the cycle trip was synchronized with the second investigator waiting at the destination, and on arrival the total trip time was noted. The participants were asked to confirm whether their drawn routes on maps had been taken the whole way, and if not, any deviation from the originally marked route was added to the map. The overall cycling velocity was calculated based on the route distances measured with the criterion method [29] and the trip time (cf. Table 1).

### Statistical analyses

Absolute and percent differences between groups in the pre-measurement methodological studies were analyzed with Student’s paired t-tests and one-sample t-tests, respectively.

The reproducibility of the paired individual data for VO_2_ and HR between test and retest in the laboratory was calculated as absolute and relative differences, and analyzed with Student´s paired t-test as well as coefficient of variation (CV). The CV was calculated by dividing the standard deviation of the difference between the test–retest values by √2. This value (typical error) was then divided with the average of the test–retest values and multiplied by 100 [37].

The HR-VO_2_ relations based on each individual´s paired VO_2_ and HR from three submaximal work rates (model 1) plus a maximal work rate (model 2), at test and retest were described by linear regression analyses and correlation coefficients (r-values). The absolute differences in y-intercepts, slopes and r-values between test and retest were evaluated with paired Student´s t-test for each model. The absolute values for the y-intercepts, slopes and r-values at test and retest were also compared between model 1 and 2 with paired Student´s t-test, and the 95% confidence intervals for the mean values were calculated.

The reproducibility of the estimated VO_2,_ based on the regression equations from test and retest, and calculated on the basis of three levels of HR from each individual´s cycle commuting, is presented in absolute figures and absolute as well as relative differences. They were analyzed for all individuals with Student´s paired t-test, the 95% confidence intervals for the mean values and coefficient of variation (CV).

Whether the levels of estimated VO_2_ at test and retest, as well as the differences between test and retest, were altered between model 1 and 2 were also evaluated with Student´s paired t-test and 95% confidence intervals for the mean values. Bland-Altman plots with 95% limits of agreement in individual absolute values of estimated VO_2_ were graphically displayed [38].

Statistical analyses were performed using the Statistical Package for the Social Sciences (SPSS, 21.0, Chicago, IL, USA). The Bland-Altman plots were created with Graph-Pad Prism, software package version 8.1.1 (330), April 11, 2019 (Graph-Pad Software Inc., San Diego, CA, USA). Values are presented as mean ± standard deviation (SD) unless otherwise stated. The significance level was set at p<0.05 when data were used only once, and at p<0.025 when data were used twice.

## Results

### Reproducibility of repeated single measurements

There were no systematic absolute or relative differences in VO_2_ and HR between the first and second measurement occasion in the laboratory (Table 3).

**Table 3:** Test-retest of VO_2_ and HR at rest, submaximal and maximal cycle ergometer tests in the laboratory (mean ± SD and coefficient of variation, CV).

Work rates	Males (n = 9)							Females (n = 10)							
	Oxygen uptake, L · min^-1^							Work rates	Oxygen uptake, L · min^-1^						
	Test	Retest	Absolute difference, L·min^-1^	Relative difference, %	p-values abs.	p- values %	CV		Test	Retest	Absolute difference, L·min^-1^	Relative difference, %	p-values abs.	p- values %	CV
100 W	1.46 ± 0.10	1.42 ± 0.14	-0.04 ± 0.11	-2.4 ± 8.2	0.368	0.407	5.5	50 W	0.82 ± 0.05	0.84 ± 0.09	0.20 ± 0.10	2.8 ± 12.0	0.558	0.483	8.8
150 W	2.04 ± 0.09	2.03 ± 0.14	-0.01 ± 0.09	-0.6 ± 4.6	0.725	0.708	3.2	100 W	1.42 ± 0.07	1.42 ± 0.12	0.00 ± 0.15	0.2 ± 10.3	0.984	0.945	7.5
194 ± 11 W	2.66 ± 0.19	2.61 ± 0.25	-0.04 ± 0.12	-1.7 ± 4.7	0.318	0.308	3.3	140 ± 13 W	1.91 ± 0.18	1.93 ± 0.16	0.03 ± 0.10	1.7 ± 5.2	0.395	0.336	3.6
Maximal	3.91 ± 0.55	3.94 ± 0.63	0.03 ± 0.28	1.0 ± 7.3	0.730	0.705	5.0	Maximal	2.56 ± 0.33	2.56 ± 0.28	0.00 ± 0.18	0.4 ± 6.8	0.986	0.848	4.8
**Rest and work rates**	**Heart rate**, beats · min^-1^							**Rest and work rates**	**Heart rate**, beats · min^-1^						
Rest	61 ± 8	58 ± 10	-2.78 ± 5.56	-4.7 ± 9.2	0.172	0.163	6.6	Rest	63 ± 7	65 ± 8	2.40 ± 6.08	4.1 ± 9.8	0.243	0.222	6.7
100 W	97 ± 8	98 ± 9	0.11 ± 4.34	0.1 ± 4.4	0.941	0.975	3.1	50 W	98 ± 8	99 ± 10	1.10 ± 7.75	1.3 ± 7.6	0.664	0.615	5.6
150 W	118 ± 15	116 ± 11	-2.33 ± 7.73	-1.5 ± 5.8	0.392	0.446	4.7	100 W	124 ± 9	124 ± 10	-0.60 ± 5.66	-0.5 ± 4.4	0.745	0.740	3.2
194 ± 11 W	139 ± 18	135 ± 15	-4.28 ± 6.04	-2.8 ± 4.3	0.066	0.082	3.1	140 ± 13 W	150 ± 10	149 ± 11	-1.60 ± 2.67	-1.1 ± 1.8	0.091	0.086	1.3
Maximal	174 ± 11	173 ± 10	-1.00 ± 3.24	-0.5 ± 1.9	0.382	0.437	1.3	Maximal	180 ± 10	178 ± 10	-1.70 ± 2.79	-0.1 ± 1.5	0.086	0.080	1.1

Note: The p-values are based on the paired differences in absolute and relative terms. CV is based on the absolute values.

### Positioning work rates for the HR-VO_2_ relations in the laboratory

The three submaximal work rates, used in both models of HR-VO_2_ regression equations, induced mean levels of HR ranging from on average 97 ± 8 to 139 ± 18 beats per minute for the males, and from 98 ± 8 to 150 ± 10 for the females (Table 3). For maximal HR and other descriptive aspects of the work rates used, see Tables 3 and 4.

**Table 4:** Positions of ergometer cycle work rates used to determine the HR-VO_2_ relations in males and females and responses to them in per cent of VO_2max_, heart rate reserve and per cent of HR_max_, as well as RPE of legs and breathing during test 1 in the laboratory (mean ± SD).

	Responses to ergometer cycle work rates							
Sex	Males (n = 9)				Females (n = 10)			
**Work rates**	100 W	150 W	194 ± 11 W	Maximal	50 W	100 W	140 ± 13 W	Maximal
**Per cent of maximal oxygen uptake**	37.9 ± 5.6	53.1 ± 7.5	69.1 ± 10.2	100	32.4 ± 2.1	56.1 ± 4.9	75.0 ± 7.7	100
**Per cent of heart rate reserve**	32.2 ± 3.9	50.6 ± 9.6	68.9 ± 9.6	0	29.4 ± 4.5	52.3 ± 4.7	74.3 ± 7.4	0
**Per cent of maximal heart rate**	56.1 ± 2.1	68.0 ± 5.8	79.9 ± 6.1	100	54.2 ± 3.6	69.1 ± 2.9	83.4 ± 4.7	100
**Rated perceived exertion, legs**	11.1 ± 1.3	12.7 ± 1.2	15.0 ± 1.1	18.4 ± 1.0	9.3 ± 1.7	12.5 ± 0.8	14.8 ± 0.9	17.8 ± 1.0
**Rated perceived exertion, breathing**	11.6 ± 1.2	12.8 ± 1.9	14.6 ± 1.7	18.3 ± 1.2	9.1 ± 1.7	12.4 ± 1.1	14.5 ± 1.0	18.8 ± 1.1

### HR levels from commuter cycling used for estimating levels of VO_2_

The mean values of the 20% lowest, intermediate and highest heart rate segments during the commuter cycling and their mean HR values are described in Table 5.

**Table 5:** The three HR levels from cycle commuting used to estimate VO_2_ based on the HR-VO_2_ regression equations at test and retest. The corresponding levels of percent of heart rate reserve and percent of HR_max_ are also given (mean ± SD).

	Heart rates during cycle commuting					
Sex	Males (n = 9)			Females (n = 10)		
**HR segments**, %	0–20	41–60	81–100	0–20	41–60	81–100
**Heart rate**, beats/minute	113 ± 10	136 ± 11	149 ± 10	118 ± 10	139 ± 6	154 ± 7
**Percent of heart rate reserve**	47.4 ± 9.9	67.5 ± 12.2	78.5 ± 11.9	50.4 ± 7.1	68.5 ± 4.6	81.0 ± 4.6
**Percent of maximal heart rate**	65.5 ± 6.8	78.5 ± 8.3	85.8 ±7.9	67.2 ± 5.6	79.2 ± 3.8	87.4 ± 3.4

Note: The percent of heart rate reserve and HR_max_ are based on HR at rest and at maximal efforts on a cycle ergometer in the laboratory 15 ± 10 days prior to the field tests.

The mean levels of all HR (not shown) were somewhat lower than the intermediate 1/5 of the size ordered HR. This is since the lowest 1/5 of HR is clearly further away from the intermediate 1/5 than the distance to the highest 1/5 of HR.

### Reproducibility of HR-VO_2_ regression equations and estimated levels of VO_2_ (model 1)

**Fig 2:**
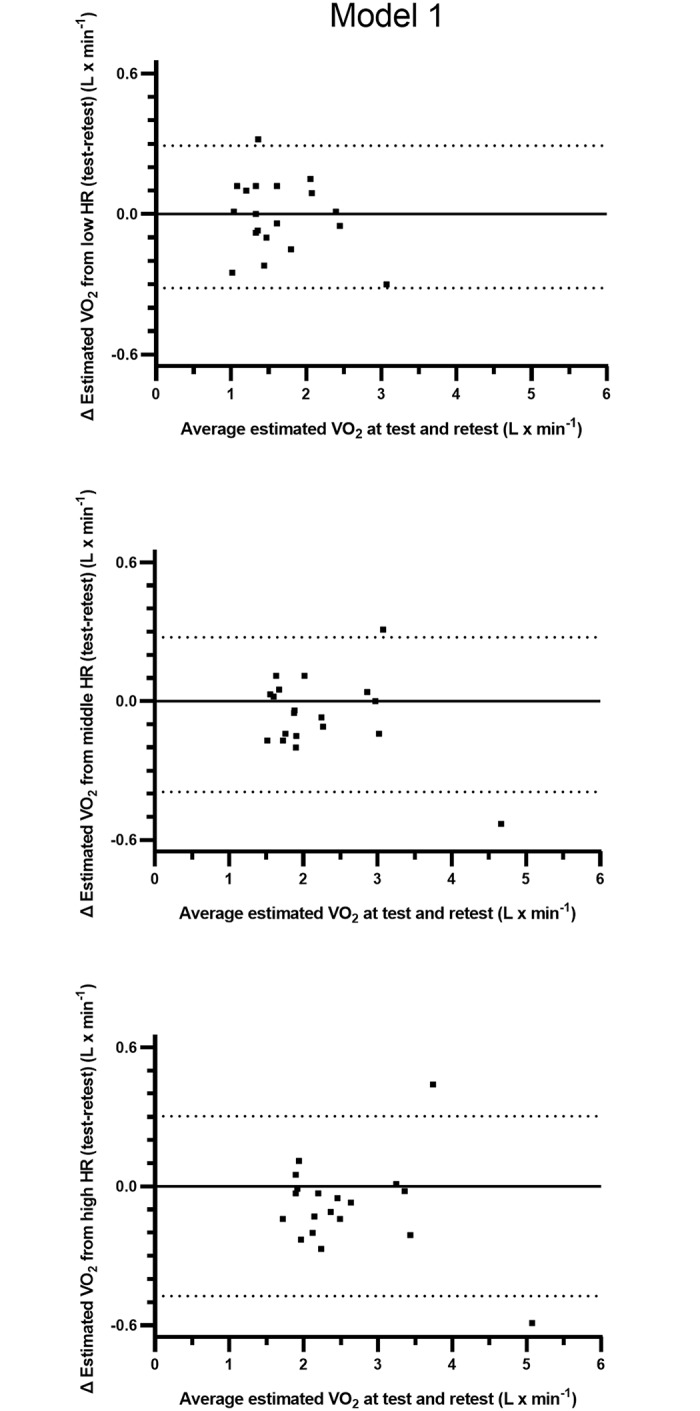
Individual levels of differences and the 95% limits of agreement between estimations of VO_2_ (L · min^-1^) calculated from fixed levels of low, middle and high HR from commuter cycling as well as from repeated measurements of HR-VO_2_ relations based on three submaximal work rates (model 1). The y-axes show absolute differences in VO_2_ against the mean values of the estimations from the repeated measurements on the x-axes.

The test and retest HR-VO_2_ regression equations and estimated levels of oxygen uptake from three levels of HR are presented in Tables 6 and 7. There was a tendency towards a lower y-intercept and a greater slope in the regression equations at the retest compared to the test (Table 6). Based on calculations of all subjects, there were no systematic differences in estimated absolute levels of VO_2_ between test and retest. The relative differences between test and retest were 0.99 ± 11.0 (n.s.), 2.67 ± 6.48 (p<0.1) and 3.57 ± 6.24% (p<0.05) based on estimations from the lowest to the highest levels of HR (Table 7). The individual data for all tables (Tables 6–11) related to evaluations of the HR-VO_2_ relations are given as Supporting Information S1 Results. The 95% limits of agreement for the individual variations in the differences in estimated VO_2_ between test and retest varied between -0.3155 and 0.2923) (L · min^-1^) for the low HR, -0.3922 and 0.2764 for the middle HR, and -0.4735 and 0.3029 for the high HR (Fig 2).

**Table 6:** Reproducibility of HR-VO_2_ regression equations and correlation coefficients based on three submaximal work rates (model 1)(means ± SD, n = 19).

	HR-VO_2_ regression equations					
	Day 1			Day 2		
	y-intercept	slope	r	y-intercept	slope	r
Mean	-1.33	0.0255	0.996	-1.58	0.0277	0.997
SD	0.59	0.0080	0.005	0.74	0.0089	0.004
P-value abs. diff. day 1 and 2				.085	.053	.560

**Table 7:** The estimated levels of VO_2_ based on the regression equations in day 1 and 2 (model 1) and three levels of HR from cycle commuting (means ± SD, coefficients of variation (CV), and 95% confidence intervals (CI), n = 19).

	HR at field and estimations of VO_2_ based on three levels of HR and the HR-VO_2_ regression equations at day 1 and 2														
	Lowest fifth of HR					Middle fifth of HR					Highest fifth of HR				
	HR F1	VO_2_:1	VO_2_:2	Abs diff	% diff	HR F3	VO_2_: 1	VO_2_:2	Abs diff	% diff	HR F5	VO_2_:1	VO_2_:2	Abs diff	% diff
Mean	116	1.63	1.64	0.01	0.99	138	2.19	2.25	0.06	2.67	151	2.53	2.61	0.08	3.57
SD	10.0	0.54	0.57	0.15	11.0	8.49	0.76	0.82	0.17	6.48	8.27	0.83	0.88	0.20	6.24
P-value				.749	.699				.153	.089				.079	.022
CI lower				-0.06	-4.29				-0.02	-0.45				-0.01	0.57
CI upper				0.09	6.27				0.14	5.79				0.18	6.58
CV				6.67					5.39					5.45	

**Table 8:** Reproducibility of HR-VO_2_ regression equations and correlation coefficients based on three submaximal and a maximal work rate (model 2)(means ± SD, n = 19).

	HR-VO_2_ regression equations					
	Day 1			Day 2		
	y-intercept	slope	r	y-intercept	slope	r
Mean	-1.44	0.0263	0.993	-1.55	0.0273	0.996
SD	0.68	0.0089	0.013	0.83	0.0098	0.009
P-value abs. diff. day 1 and 2				.375	.277	.472

**Table 9:** The estimated levels of VO_2_ based on the HR-VO_2_ regression equations in day 1 and 2 (model 2) and three levels of HR from cycle commuting (means ± SD, coefficients of variation (CV), and 95% confidence intervals (CI), n = 19).

	HR at field and estimations of VO_2_ based on three levels of HR and the HR-VO_2_ regression equations at day 1 and 2														
	Lowest fifth of HR					Middle fifth of HR					Highest fifth of HR				
	HR F1	VO_2_:1	VO_2_:2	Abs diff	% diff	HR F3	VO_2_: 1	VO_2_:2	Abs diff	% diff	HR F5	VO_2_:1	VO_2_:2	Abs diff	% diff
Mean	116	1.62	1.63	0.01	1.09	138	2.21	2.24	0.03	1.75	151	2.55	2.60	0.05	2.12
SD	10.0	0.57	0.60	0.14	10.6	8.49	0.83	0.88	0.15	6.43	8.27	0.90	0.94	0.16	5.92
P-value				.769	.661				.321	.252				.219	.136
CI lower				-0.06	-4.04				-0.04	-1.35				-0.03	-0.74
CI upper				0.08	6.22				0.11	4.85				0.13	4.97
CV				6.25					4.72					4.44	

**Table 10:** Differences between model 1 and model 2 in the HR-VO_2_ regression equations and correlation coefficients (means ± SD, and 95% confidence intervals (CI), n = 19).

	Differences between model 1 and 2 in the constituents of HR-VO_2_ regression equations and the correlation coefficient at day 1 and 2					
	Day 1			Day 2		
	y-intercept	slope	r	y-intercept	slope	r
Mean	-0.10	0.0008	-0.003	0.03	-0.0003	0.002
SD	0.42	0.0037	0.012	0.35	0.0032	0.009
P-value	.297	.327	.244	.672	.647	.403
CI lower	-0.30	-0.001	-0.009	-0.13	-0.0018	-0.006
CI upper	0.10	0.003	0.002	0.20	0.0012	0.003

**Table 11:** Differences between model 1 and model 2 in the estimated VO_2_ based on three levels of HR as well as in absolute and relative differences (means ± SD, and 95% confidence intervals (CI), n = 19).

	Differences between model 1 and 2 in estimations of VO_2_ based on the HR-VO_2_ regression equations at day 1 and 2											
	Lowest fifth of HR				Middle fifth of HR				Highest fifth of HR			
	VO_2_:1	VO_2_:2	Abs diff	% diff	VO_2_:1	VO_2_:2	Abs diff	% diff	VO_2_:1	VO_2_:2	Abs diff	% diff
Mean	0.00	-0.01	0.00	0.10	0,02	-0,01	-0,02	-0,92	0.03	-0.01	-0.04	-1.46
SD	0.07	0.04	0.04	3.39	0,12	0,10	0,07	2,82	0.15	0.13	0.10	3.30
P-value	.790	.532	.866	.900	.553	.759	.160	.171	.449	.723	.125	.071
CI lower	-0.04	-0.03	-0.02	-1.54	-0.04	-0.05	-0.06	-2.28	-0.05	-0.07	-0.09	-3.05
CI upper	0.03	0.01	0.02	1.74	0.07	0.04	0.01	0.44	0.10	0.05	0.01	0.14

### Reproducibility of HR-VO_2_ regression equations and estimated levels of VO_2_ (model 2)

**Fig 3:**
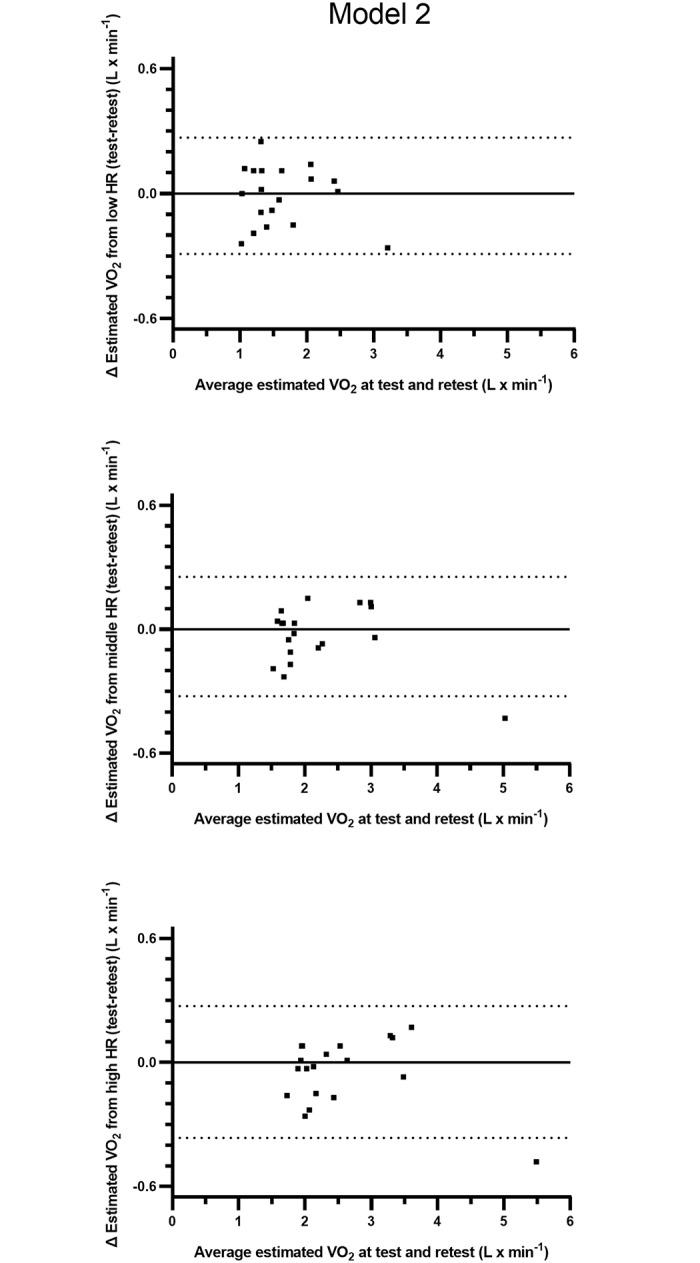
Individual levels of differences and the 95% limits of agreement between estimations of VO_2_ (L · min^-1^) calculated from fixed levels of low, middle and high HR from commuter cycling as well as from repeated measurements of HR-VO_2_ relations based on three submaximal work rates (model 2). The y-axes show absolute differences in VO_2_ against the mean values of the estimations from the repeated measurements on the x-axes.

The test and retest HR-VO_2_ regression equations and estimated levels of oxygen uptake from three levels of HR are presented in Tables 8 and 9. There were no significant differences between test and retest in the constituents of the regression equations (y-intercept, slope and r-value)(Table 8). Based on calculations of all subjects, there were no systematic differences in estimated absolute levels of oxygen uptake between test and retest. The relative differences between test and retest, based on estimations from three different levels of HR, were 1.09 ± 10.6, 1.75 ± 6.43 and 2.12 ± 5.92% (all n.s.)(Table 9). The 95% limits of agreement for the individual variations in the differences in estimated VO_2_ between test and retest varied between -0.2894 and 0.2684)(L · min^-1^) for the low HR, -0.3233 and 0.2539 for the middle HR, and -0.3649 and 0.2722 for the high HR (Fig 3).

### Comparisons in regression equations and estimated VO_2_ between the HR-VO_2_ relations in model 1 and 2

The differences between the two HR-VO_2_ models in the y-intercept, slope, r-value as well as in the three levels of estimated VO_2_ at test and retest were compared for all subjects (Tables 10 and 11). All differences between the models were small and non-significant. The mean absolute and relative differences in VO_2_ varied from 0.00 ± 0.04 to -0.04 ± 0.10 liter/min (all n.s.) and 0.10 ± 3.39 to -1.46 ± 3.30% (all n.s.), respectively.

## Discussion

An important feature of this study is that we have developed a transparent framework for analyses of the reproducibility of the HR method in laboratory conditions. It is characterized by positioning all HR values used in relation to both resting and maximal HR. This relates to both the HR-VO_2_ relations that were established in the laboratory, and the evaluation of them with three relevant HR levels that were obtained from cycle commuting in field conditions. In this way, the relative localisation of the measurement points of HRs used is clarified in a way that can be reproduced, and compared with future studies of these matters.

The main finding of the study is the absence of significant differences between test and retest in the constituents of the regression equations (y-intercept, slope and r-value) in model 2, which is constructed with three submaximal and a maximal work rate. In line with this, the estimations of VO_2_, based on three levels of HR and HR-VO_2_ regression equations from submaximal and maximal work rates (model 2), were stable at the group level. The range of the average relative differences in estimated VO_2_ was 1.09–2.12% (n.s.). However, the individual day-to-day variations can be of greater magnitude, as indicated by the range of standard deviations for the relative differences (5.92–10.6%). Consequently, the 95% confidence intervals for the mean values of all subjects indicate variations of between approximately 6–10% for the three different estimations of relative differences in VO_2_ between test and retest. This spreading is further illustrated in the individual differences between test and retest, and in the 95% limits of agreements (cf. Fig 3).

The results with model 1, based on only three submaximal work rates, were essentially the same, and in the same order of magnitude (cf. Fig 2). However, there was a tendency towards small differences between test and retest in the constituents of the regression equations (y-intercept and slope), and based on the highest fifth of HR from the cycle commuting, the relative difference in estimated VO_2_ was 3.57% (p<0.05) higher at retest.

Another important finding was an absence of significant differences between model 1 and 2 in the constituents of the regression equations and in the estimated levels of VO_2_.

The first issue to be noted is the overall pattern of stability on the group levels between test and retest in VO_2_ and HR, which permits the present test-retest analyses of the outcomes of the HR-VO_2_ regression equations. The fact that we started the measurements with 15 minutes of rest in a supine position, and that all subjects were very physically active (cf. Table 1) and familiar with the cycling movement as cycle commuters, can be important for this outcome. It should, at the same time, be kept in mind that habituation effects in HR with repeated measurements have been noted in studies of samples from the general population [20,22,23]. As a safeguard, a habituating pre-test, as was applied by McCrory et al. [25], is therefore recommended as a standard procedure.

Non-systematic test-retest variability in HR was also noted by McCrory et al. [25]. They even controlled for sex and some individual factors, such as being an emotional person, but without being able to see any such relations. Berggren and Hohwü-Christensen [8] studied the HR-VO_2_ relation repeatedly in one person, and found variability in the HR of the same order of absolute magnitude in work rates demanding between 1 to 4 litres of VO_2_. Thus, it is possible that this variability stands for an intrinsic feature of repeated HR-VO_2_ spot measures. In many ways, this is reasonable, since the VO_2_, according to the Fick principle, is the product of heart rate, stroke volume and the difference between arterial and mixed venous oxygen content. Thus, levels of four different variables can vary in response to a work load, and still the resulting VO_2_ can be the same. From that perspective, it is not surprising that the HR may vary from time to time at a given exercise-induced VO_2_. It indicates that the biological steering mechanisms for these variables might not be strictly controlled.

In individual cases, linearity between HR and VO_2_ has been indicated to sometimes end at near to maximal VO_2_ levels, with greater increases in VO_2max_ than in HR [7, 39], (p. 352 in [40]). Given that, it could be questionable to include values on maximal HR and VO_2_, as we did in model 2 in this study, and therefore it could be anticipated that the regression equations and outcomes of model 1 and 2 might differ. Including maximal HR and VO_2_ could, on the other hand, serve as an anchor, stabilizing effects of day to day variability of the regression equations that otherwise could come into play. One reason for such a role for HR values from maximal work rate is its low CV (Table 3) in comparison with those at the submaximal work rates. The fact that we did not see any significant differences between the outcomes in model 1 and 2 indicates the potential value of educational or clinical models that do not include measurements from maximal work rates. Furthermore, it also indicates that research models for establishing the HR-VO_2_ relation may be adequate without maximal measurements. Adding more submaximal measurements than those three that we have used, might, however, be a fruitful way to create even greater day to day stability in models based on only submaximal work rates. This deserves future studies.

One reason for the good reproducibility on the group level for model 1, despite only making use of three submaximal work rates, can be the span of the HR attained between work rate 1 and 3 (in average 98–137 and 98–150 for males and females, respectively). It is equally important that the utilized ranges of HR from cycle commuting (in average 113–149 and 118–154 for males and females, respectively) are within, or only slightly above, the range of the HR from the work rates in the laboratory (cf. Tables 3 and 5). If instead VO_2_ would be estimated from higher or lower HR than those established in the laboratory, it is possible that greater test-retest differences would be seen (cf. Fig 1).

A comment on the field heart rates used is that almost half the cyclists were tested in the laboratory at a different time of day compared to their cycle commuting tests. However, McCrory et al. [25] found that within-day variations were not significantly different at the higher activity levels in their study, i.e. normal to fast walking. Thus our population probably had levels of intensity that were high enough to eliminate circadian influences. Another comment favoring a stability in the measurement conditions is that the mean values for the positions of % HR_max_ used to establish the HR-VO_2_ relations related well to the expected VO_2_ relative to VO_2max_ in both sexes [26].

Our results are in line with those of McCrory et al. [25], and considerably more favourable in relation to using the HR-VO_2_ method than those indicated by Christensen et al. [24]. There are several explanations for that. The measurements used by Christensen et al. [24] for establishing HR-VO_2_ regression equations were resting and sitting, as well as three low to intermediate exercise rates on an ergometer cycle (8–100 watt) and three exercise rates on a treadmill, thus altogether eight measurement points. For both the slope and the y-intercept of the regression equations, the measurements at low levels of HR are, under those circumstances, more influential. At the same time it is well known that the HR-VO_2_ ratios at rest and sitting are quite unstable, resulting in variations in regression equations [11, 25, 41]. Between very low intensities of exercise and rest, the slope of the linear relationship between VO_2_ and HR will be higher after a certain HR level, which has been termed “flex HR” [12, 42, 43], which could be another reason for the results of Christensen et al. [24]. Furthermore, they mixed the work forms of cycle ergometer and treadmill as bases for the HR-VO_2_ measures, which is in itself problematic, since the HR response for a given VO_2_ can differ in these different forms of movement [44]. This creates a greater risk for non-stability in regression equations with repeated measurements. Finally, the measures of 24-hour HR by Christensen et al. [24] resulted in a mean value of 86 beats per minute. In line with the reasoning in the Introduction (cf. Fig 1) a heart rate close to the endpoint of the spectrum of measurement points forming the regression equation will most probably lead to lower reproducibility. Another potential explanation for their results relates to their use of a heterogeneous sample of predominantly patients and large variations in age, whereas we studied a sample of healthy and physically active middle aged individuals.

Having stated that, one has to keep in mind that the external validity of our findings in relation to other types of participants is uncertain. Thus, to forward the general knowledge in these respects, there is indeed a need for further studies of these matters.

Furthermore, we do not know anything about the external validity of the HR method in the laboratory in relation to field conditions such as during cycle commuting. Three studies have looked at the intensity of cycle commuting using different HR methods in samples of non-regular cycle commuters [45, 46, 47]. However, none of these studies considered that, for reasons such as cardiovascular drift with prolonged work durations [12, 48, 49] or stress due to traffic conditions [50, 51], the relationship measured in the laboratory may differ when being in a cycle commuting environment, and that consequently the indicated intensity of cycle commuting might be incorrect. This will be the focus in our further studies.

We have, as pointed out in the beginning of the Discussion, developed a framework for studying these matters in terms of relating all HR used to the maximal HR (%maxHR) and the relative position of the HR in between the resting and the max HR (%HRR). In future studies we do also suggest that the body temperature is monitored, since this factor influences the metabolism and may affect the blood flow distribution and thereby also the constituents of the Fick principle, with possible effects on HR-VO_2_ relations.

In conclusion, this study has demonstrated a good reproducibility on the group level for two models of HR-VO_2_ relations that were established through cycle ergometer exercise in laboratory conditions with healthy and physically active middle-aged participants, and evaluated with three levels of HR that are representative of moderate exercise intensities. However, on the individual level some rather large variations were noted.

## Supporting information

S1 Methods The Physically Active Commuting in Greater Stockholm Questionnaire 1 (PACS Q1). The original version in Swedish.
(DOC)

S2 Methods The Physically Active Commuting in Greater Stockholm Questionnaire 1 (PACS Q1). The original version in Swedish translated into English.
(DOC)

S3 Methods Instructions for how to fill in the Physically Active Commuting in Greater Stockholm Questionnaire 1 (PACS Q1) and maps. The original version in Swedish.
(DOC)

S4 Methods Instructions for how to fill in the Physically Active Commuting in Greater Stockholm Questionnaire 1 (PACS Q1) and maps. The original version in Swedish, translated into English.
(DOC)

S5 Methods Information about the exercise physiological tests, standardization demands and a medical enquiry. The original version in Swedish.
(DOC)

S6 Methods Information about the exercise physiological tests, standardization demands and a medical enquiry. The original version in Swedish, translated into English.
(DOC)

S7 Methods The Physically Active Commuting in Greater Stockholm Questionnaire 2 (PACS Q2). The original version in Swedish.
(DOC)

S8 Methods The Physically Active Commuting in Greater Stockholm Questionnaire 2 (PACS Q2). The original version in Swedish translated into English.
(DOCX)

S1 Results The individual data, grouped by sex, that constitute the bases for Tables 6–11. (RTF)
