# Supplementary material for: The heart rate method for estimating oxygen uptake: Analyses of reproducibility using a range of heart rates from cycle commuting
Source: PLoS One. 2019 Jul 24;14(7):e0219741. doi: 10.1371/journal.pone.0219741 (PMC6655643; doi:10.1371/journal.pone.0219741)
Supplement: S1 Methods — The original version in Swedish. (DOC) [file pone.0219741.s001.doc]

### Enkät till Dig som går eller cyklar hela vägen till arbets-/studieplatsen

#### Allmänna frågor

1. **Är Du kvinna eller man?**  Kvinna  Man
2. **Vilket år föddes Du?** 19
3. **Hur mycket väger Du?** Svara i hela kilo  kg
4. **Hur lång är Du?**  cm
5. **Är Du**: **förvärvsarbetande?**  **frivilligarbetande?**  **studerande?**  **sysselsatt med annat?,**  ange vad:……………….…..
6. **Har Du tillgång till dusch på arbets-/studieplatsen?**

    Ja, på ett smidigt sätt  Ja, men inte på ett smidigt sätt  Nej  Vet ej

Du som går hela vägen till arbets-/studieplatsen, men aldrig cyklar dit kan gå direkt till fråga 18 på sidan 5.

| 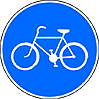 | Frågor om din cykeltur till arbets-/studieplatsen | 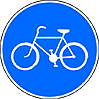 |
| --- | --- | --- |

1. **Vilken är din vanligaste färdväg till och från arbets-/studieplatsen?** Markera på den karta du fått. Följ bifogat instruktionsbrev.
2. **Uppskatta hur lång din färdväg är.** Ange ungefärligt avstånd i kilometer, gärna med en decimal (t.ex. 600 meter = 0,6 km).

   ,  km
3. **Hur lång tid tar vanligtvis cykelturen från bostaden till arbets-/studieplatsen?**Ta tid på färden en vanlig dag då du inte gör ärenden på vägen.  Timmar  Minuter
4. **Hur lång tid tar vanligtvis cykelturen från arbets-/studieplatsen till bostaden?**
   Ta tid på färden en vanlig dag då du inte gör ärenden på vägen.
    Timmar  Minuter
5. **Hur ansträngande i genomsnitt upplever du att cykelturen till arbets-/studieplatsen vanligtvis är?** Sätt ett kryss i varje kolumn vid någon siffra
   i skalan 6-20.

**Från bostaden till arbets-/studieplatsen**

 6
 7 Mycket mycket lätt
 8
 9 Mycket lätt
 10
 11 Lätt
 12
 13 Något ansträngande
 14
 15 Ansträngande
 16
 17 Mycket ansträngande
 18
 19 Mycket mycket ansträngande
 20

**Från arbets-/studieplatsen till bostaden**

6
 7 Mycket mycket lätt
 8
 9 Mycket lätt
 10
 11 Lätt
 12
 13 Något ansträngande
 14
 15 Ansträngande
 16
 17 Mycket ansträngande
 18
 19 Mycket mycket ansträngande
 20

1. **Cyklar du vanligtvis till och hem från arbets-/studieplatsen under samma dag?** Det vill säga du låter inte cykeln stå kvar på arbets-/studieplatsen.

     Ja  Nej  Vet ej

Om du svarat Nej ange gärna varför: …………………………………………………… ….…………………………………………………………………………..………………..

Nu kommer en fråga som tillsammans med de färdvägar som du ritar in på kartan kommer att ge en värdefull bild av din fysiska aktivitet och även av möjliga hälsoeffekter av cyklingen.

1. **Hur många cykelturer (se instruktion nedan) gör du mellan bostaden och arbets-/studieplatsen i medeltal per vecka under olika månader?** Sätt ett kryss för varje månad.

***Så här fyller du i dina svar:***

- **Om du cyklar till och från arbets-/studieplatsen 5 dagar i veckan under hela månaden blir antalet cykelturer i medeltal 10 per vecka.**
- **Om du istället är ledig halva månaden blir antalet cykelturer i medeltal 5 per vecka under den månaden.**
- **Om du har semester hela månaden blir antalet cykelturer 0.**
- **Om du i medeltal gör färre än 1 cykeltur i veckan men sammanlagt fler än 0 ska du fylla i rutan för <1.**
- **Om du cyklar till och/eller från arbets-/studieplatsen oregelbundet och vid ett fåtal tillfällen över året samt är osäker på vilka månader som du gör det kryssar du i rutan ”*Annat alternativ*”.**

| **Månad** | Antal cykelturer i medeltal per vecka | | | | | | | | | | | | | | | | | | |
| --- | --- | --- | --- | --- | --- | --- | --- | --- | --- | --- | --- | --- | --- | --- | --- | --- | --- | --- | --- |
|  | 0 | <1 | 1 | 2 | 3 | 4 | 5 | 6 | 7 | 8 | 9 | 10 | 11 | 12 | 13 | 14 | Fler... | ange antal: | Vet ej |
| **januari** |  |  |  |  |  |  |  |  |  |  |  |  |  |  |  |  |  | ….. |  |
| **februari** |  |  |  |  |  |  |  |  |  |  |  |  |  |  |  |  |  | ….. |  |
| **mars** |  |  |  |  |  |  |  |  |  |  |  |  |  |  |  |  |  | ….. |  |
| **april** |  |  |  |  |  |  |  |  |  |  |  |  |  |  |  |  |  | ….. |  |
| **maj** |  |  |  |  |  |  |  |  |  |  |  |  |  |  |  |  |  | ….. |  |
| **juni** |  |  |  |  |  |  |  |  |  |  |  |  |  |  |  |  |  | ….. |  |
| **juli** |  |  |  |  |  |  |  |  |  |  |  |  |  |  |  |  |  | ….. |  |
| **augusti** |  |  |  |  |  |  |  |  |  |  |  |  |  |  |  |  |  | ….. |  |
| **september** |  |  |  |  |  |  |  |  |  |  |  |  |  |  |  |  |  | ….. |  |
| **oktober** |  |  |  |  |  |  |  |  |  |  |  |  |  |  |  |  |  | ….. |  |
| **november** |  |  |  |  |  |  |  |  |  |  |  |  |  |  |  |  |  | ….. |  |
| **december** |  |  |  |  |  |  |  |  |  |  |  |  |  |  |  |  |  | ….. |  |

**Annat alternativ …………………..**

1. **Hur många gånger behöver du vanligtvis stanna för rött ljus under färden till arbets-/studieplatsen?** Räkna gärna antalet stopp du gör under en vanlig dag.

    Stannar ej
    Stannar. Ange antal stopp:

     1  2  3  4  5  6  7  8  9  10  11  12  13  14  15  16  17  18  19  20  Mer än 20 gånger, ange antal……  Vet ej
2. **Blir du svettig när du cyklar till arbets-/studieplatsen?**

Nej, aldrig
 Ja, 1-25 % av gångerna
 Ja, 26-50 % av gångerna
 Ja, 51-75 % av gångerna
 Ja, 76-100 % av gångerna
 Vet ej

1. **Brukar du duscha efter cykelturen till arbets-/studieplatsen?**

Nej, aldrig
 Ja, 1-25 % av gångerna
 Ja, 26-50 % av gångerna
 Ja, 51-75 % av gångerna
 Ja, 76-100 % av gångerna
 Vet ej

#### Fråga om din cykel

1. **Vilken typ av cykel använder du på cykelturen till arbets-/studieplatsen?**  Oväxlad cykel
    Växlad cykel (2- 4 växlar)
    Växlad cykel (5 växlar eller fler)
    Vet ej

Fortsätt med nästa fråga om du under det senaste året vid något tillfälle gått hela vägen till din arbets-/studieplats. Fortsätt annars med fråga 28 på sidan 8.

| 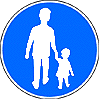 | Frågor om din gångtur till arbets-/studieplatsen | 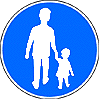 |
| --- | --- | --- |

1. **Vilken är din vanligaste färdväg till och från arbets-/studieplatsen?** Markera på den karta du fått. Följ bifogat instruktionsbrev.

1. **Uppskatta hur lång din färdväg är.** Ange ungefärligt avstånd i kilometer, gärna med en decimal (t.ex. 600 meter = 0,6 km)

   ,  km
2. **Hur lång tid tar vanligtvis gångturen från bostaden till arbets-/studieplatsen?**Ta tid på färden en vanlig dag då du inte gör ärenden på vägen.  Timmar  Minuter
3. **Hur lång tid tar vanligtvis gångturen från arbets-/studieplatsen till bostaden?**
   Ta tid på färden en vanlig dag då du inte gör ärenden på vägen.
    Timmar  Minuter
4. **Hur ansträngande i genomsnitt upplever du att gångturen till arbets-/studieplatsen vanligtvis är?** Sätt ett kryss i varje kolumn vid någon siffra
   i skalan 6-20.

**Från bostaden till arbets-/studieplatsen**

 6
 7 Mycket mycket lätt
 8
 9 Mycket lätt
 10
 11 Lätt
 12
 13 Något ansträngande
 14
 15 Ansträngande
 16
 17 Mycket ansträngande
 18
 19 Mycket mycket ansträngande
 20

**Från arbets-/studieplatsen till bostaden**

6
 7 Mycket mycket lätt
 8
 9 Mycket lätt
 10
 11 Lätt
 12
 13 Något ansträngande
 14
 15 Ansträngande
 16
 17 Mycket ansträngande
 18
 19 Mycket mycket ansträngande
 20

1. **Går du vanligtvis till och hem från arbets-/studieplatsen under samma dag?**

     Ja  Nej  Vet ej

Om du svarat Nej ange gärna varför:……………………………………….…………… ………………………………………………………………………………………………

Nu kommer en fråga som tillsammans med de färdvägar som du ritar in på kartan kommer att ge en värdefull bild av din fysiska aktivitet och även av möjliga hälsoeffekter av gåendet.

1. **Hur många gångturer (se instruktion nedan) gör du mellan bostaden och arbets-/studieplatsen i medeltal per vecka under varje månad?** Sätt ett kryss för varje månad.

***Så här fyller du i dina svar:***

- **Om du går till och från arbets-/studieplatsen 5 dagar i veckan under hela månaden blir antalet gångturer i medeltal 10 per vecka.**
- **Om du istället är ledig halva månaden blir antalet gångturer i medeltal 5 per vecka under den månaden.**
- **Om du har semester hela månaden blir antalet gångturer 0.**
- **Om du i medeltal gör färre än 1 gångtur i veckan men sammanlagt fler än 0 ska du fylla i rutan för <1.**
- **Om du går till och/eller från arbets-/studieplatsen oregelbundet och vid ett fåtal tillfällen över året samt är osäker på vilka månader som du gör det kryssar du i rutan ”*Annat alternativ*”.**

| **Månad** | Antal gångturer i medeltal per vecka | | | | | | | | | | | | | | | | | | |
| --- | --- | --- | --- | --- | --- | --- | --- | --- | --- | --- | --- | --- | --- | --- | --- | --- | --- | --- | --- |
|  | 0 | <1 | 1 | 2 | 3 | 4 | 5 | 6 | 7 | 8 | 9 | 10 | 11 | 12 | 13 | 14 | Fler… | ange antal: | Vet ej |
| **januari** |  |  |  |  |  |  |  |  |  |  |  |  |  |  |  |  |  | …... |  |
| **februari** |  |  |  |  |  |  |  |  |  |  |  |  |  |  |  |  |  | …... |  |
| **mars** |  |  |  |  |  |  |  |  |  |  |  |  |  |  |  |  |  | …... |  |
| **april** |  |  |  |  |  |  |  |  |  |  |  |  |  |  |  |  |  | …... |  |
| **maj** |  |  |  |  |  |  |  |  |  |  |  |  |  |  |  |  |  | …... |  |
| **juni** |  |  |  |  |  |  |  |  |  |  |  |  |  |  |  |  |  | …... |  |
| **juli** |  |  |  |  |  |  |  |  |  |  |  |  |  |  |  |  |  | …... |  |
| **augusti** |  |  |  |  |  |  |  |  |  |  |  |  |  |  |  |  |  | …... |  |
| **september** |  |  |  |  |  |  |  |  |  |  |  |  |  |  |  |  |  | …... |  |
| **oktober** |  |  |  |  |  |  |  |  |  |  |  |  |  |  |  |  |  | …... |  |
| **november** |  |  |  |  |  |  |  |  |  |  |  |  |  |  |  |  |  | …... |  |
| **december** |  |  |  |  |  |  |  |  |  |  |  |  |  |  |  |  |  | …... |  |

Annat alternativ …………………

1. **Hur många gånger behöver du vanligtvis stanna för rött ljus under färden till arbets-/studieplatsen?** Räkna gärna antalet stopp du gör under en vanlig dag.
    Stannar ej
    Stannar. Ange antal stopp:
     1  2  3  4  5  6  7  8  9  10  11  12  13  14  15  16  17  18  19  20  Mer än 20 gånger ange antal…….  Vet ej
2. **Blir du svettig när du går till arbets-/studieplatsen?**

Nej, aldrig
 Ja, 1-25 % av gångerna
 Ja, 26-50 % av gångerna
 Ja, 51-75 % av gångerna
 Ja, 76-100 % av gångerna
 Vet ej

1. **Brukar du duscha efter gångturen till arbets-/studieplatsen?**

Nej, aldrig
 Ja, 1-25 % av gångerna
 Ja, 26-50 % av gångerna
 Ja, 51-75 % av gångerna
 Ja, 76-100 % av gångerna
 Vet ej

#### Frågor om din fysiska aktivitet under arbets-/studietid

1. **Hur fysiskt ansträngande har ditt dagliga arbete eller din dagliga sysselsättning (ej fritid) varit under de senaste 12 månaderna?**

    Mycket lätt, övervägande stillasittande

    Lätt fysiskt arbete men där jag rör mig en hel del (t.ex. lätt industriarbete, affärsbiträde, lärare)

    Ganska fysiskt ansträngande arbete (t.ex. lokalvårdare, brevbärare, sjukvårdsbiträde)

    Mycket fysiskt ansträngande arbete (tungt kroppsarbete, t.ex. cykelbud, tyngre skogsarbete eller byggnadsarbete)
2. **Har du möjlighet att motionera/träna på betald arbetstid**?

    Nej
    Ja, men utnyttjar det ej
    Ja, och jag utnyttjar det
    Vet ej

Om du svarat ”**Ja, och jag utnyttjar det**” fortsätt med nästa fråga, i andra fall fortsätt till fråga 32 på sidan 10.

1. **Vilken typ av aktivitet, samt hur ofta och hur länge motionerar/tränar du vanligtvis på betald arbetstid?** Använd ett medelvärde om det varierar mellan veckorna. Du kan ange flera aktiviteter.
    **Antal tillfällen Tid per** **Aktivitet per vecka träningspass**

    Styrketräning …………… gånger …….. min

    Konditionsträning …………… gånger …….. min

    Bollspel …………… gånger …….. min

    Motionsgymnastik …………… gånger …….. min
   (t.ex. aerobics/Friskis & Svettis)

    Annat,
   ange vad:
   ………………………..… …………… gånger …….. min
   ………………………..… …………… gånger …….. min ………………………..… …………… gånger …….. min

1. **Med vilken genomsnittlig ansträngningsnivå motionerar/tränar du vanligtvis på betald arbetstid**? Sätt endast ett kryss. Använd således ett medelvärde för de olika aktiviteterna om du utövar flera aktiviteter.

6
 7 Mycket mycket lätt
 8
 9 Mycket lätt
 10
 11 Lätt
 12
 13 Något ansträngande
 14
 15 Ansträngande
 16
 17 Mycket ansträngande
 18
 19 Mycket mycket ansträngande
 20

**Frågor om din fysiska aktivitet på fritiden, undantaget arbetspendling**

1. **Hur mycket har du i allmänhet rört dig eller ansträngt dig kroppsligt på din *fritid* under det senaste året? OBS! Sätt kryss för *alla* alternativ som stämmer in på dig.** Du ska inte räkna fysisk aktivitet vid arbetspendling d.v.s. att cykla och gå till arbets-/studieplatsen.

a) Har rört mig mycket litet

b) Har rört mig mycket litet men ibland tagit någon enstaka promenad eller liknande

c) Har fått ”vardagsmotion” i samband med städning, att gå i trappor, trädgårdsarbete, sällskapsdans, promenad eller lättare cykelturer (bortsett från gång-/cykelturer hela vägen till arbets-/studieplatsen), att man går ut med hunden etc.

d) Har, utöver aktiviteterna i c), ägnat mig åt lättare form av motion som promenader (eller andra aktiviteter med motsvarande ansträngning) *minst en gång per vecka*

e) Har ägnat mig åt mer ansträngande motion som t.ex. snabba promenader, joggning, simning, motionsgymnastik eller motsvarande *minst en gång per vecka*

f) Har regelbundet ägnat mig åt *hård träning eller tävling* där den fysiska
 ansträngningen varit stor, t.ex. löpning och olika bollspel

Du som fyllt i svarsalternativen e) och/eller f) i fråga 32 kan fortsätta med nästa fråga. Övriga kan fortsätta med fråga 35 på nästa sida.

1. **Du som fyllt i alternativ e) och/eller f) i fråga 32, vilken typ av aktiviteter, samt hur ofta och hur länge motionerar/tränar du vanligtvis?** Använd ett medelvärde om det varierar mellan veckorna. Du kan ange flera aktiviteter.

**Aktivitet Kategori Tid per** **Antal gånger Antal månader** **e) f) tillfälle per vecka per år**

Styrketräning   ……… min ……….... …………
 Konditionsträning   ……… min ……….... …………
 Bollspel   ……… min ……….... …………
 Motionsgymnastik   ……… min ……….... …………
(t.ex. aerobics/

Friskis & Svettis)
 Annat,
ange vad:
………………   ……… min ……….... …………
………………   ……… min ……….... …………

1. **Du som fyllt i alternativ e) respektive f) i fråga 32 och 33, med vilken genomsnittlig ansträngningsnivå motionerar/tränar du vanligtvis**?
   Sätt endast ett kryss i respektive kolumn. Använd således ett medelvärde för de olika aktiviteterna om du utövar flera aktiviteter inom alternativ e) respektive f).  **e) f)**  6
     7 Mycket mycket lätt
     8
     9 Mycket lätt
     10
     11 Lätt
     12
     13 Något ansträngande
     14
     15 Ansträngande
     16
     17 Mycket ansträngande
     18
     19 Mycket mycket ansträngande
     20
2. **Är du intresserad av att delta i studiens kommande steg?**

   Steg 2. Enkätstudiens andra del.  Ja  Nej  Vet ej

   Steg 3. Konditionstest och mätning
   av energiomsättning under färdvägen.  Ja  Nej  Vet ej
3. **Om du har synpunkter på denna undersökning och dess frågor
   så skriv dem gärna här och vid behov fortsätt på baksidan.**
   ______________________________________________________________________________________________________________________________________________________________________________________________________________________________________________________________________________________________________________________________________________________________________________________________________________________________________________________________________________________________________________________________________________________________________________________________________________

**Stort tack för hjälpen!**
