# Supplementary material for: The heart rate method for estimating oxygen uptake: Analyses of reproducibility using a range of heart rates from cycle commuting
Source: PLoS One. 2019 Jul 24;14(7):e0219741. doi: 10.1371/journal.pone.0219741 (PMC6655643; doi:10.1371/journal.pone.0219741)
Supplement: S2 Methods — The original version in Swedish translated into English. (DOC) [file pone.0219741.s002.doc]

### Survey for those who walk or bike all the way to their place of work/study

#### General questions

1. **Your gender – female or male?**  Female  Male
2. **In what year were you born**? 19
3. **What is your weight?** Please answer in full kg.  kg
4. **How tall are you?**  cm
5. **Are you**: **gainfully employed?**  **a volunteer?**  **a** **student?**  **Other?**  please specify:……………….…..
6. **Do you have access to a shower at your place of work/study?**

    Yes, conveniently  Yes, but not conveniently  No  Don’t know

If you walk all the way to your place of work/study but never use a bike to get there, proceed to question no. 18 on page 5.

| 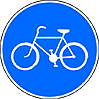 | Questions concerning your bike ride to your place of work/study | 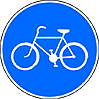 |
| --- | --- | --- |

1. **What is your usual route to and from your place of work/study?** Indicate on the map provided. Follow the attached letter of instruction.
2. **Estimate the length of your route.** Indicate the approximate distance in kilometres. You may use one decimal (e.g. 600 meters = 0.6 km).

   .  km
3. **How much time does the bike ride usually take from your home to your place of work/study?** Measure the time of the ride on an ordinary day when you do not do errands along the way.  Hours  Minutes
4. **How much time does the bike ride usually take from your place of work/study to your home?** Measure the time for the ride on an ordinary day when you do not do errands along the way.
    Hours  Minutes
5. **On average, how strenuous do you usually experience your bike ride to your place of work/study?** Mark with one x in each column at a digit on the 6 – 20 scale.

**From your home to your place of work/study**

 6
 7 Very, very light
 8
 9 Very light
 10
 11 Fairly light
 12
 13 Somewhat hard
 14
 15 Hard
 16
 17 Very hard
 18
 19 Very, very hard
 20

**From place of work/study to your home**

6
 7 Very, very light
 8
 9 Very light
 10
 11 Fairly light
 12
 13 Somewhat hard
 14
 15 Hard
 16
 17 Very hard
 18
 19 Very, very hard
 20

1. **Do you usually bike to and from your place of work/study on the same day?** I.e. you do not leave your bike at your place of work/study.

     Yes  No  Don’t know

If you answered No, explain why: ……………………………………… ….………………………………………………………………………..………………..

The following is a question, which, combined with the routes you draw on the map, will provide a valuable picture of your physical activity as well as the potential health effects of your biking.

1. **How many bike rides (see instruction below) do you make between your home and your place of work/study on average per week during different months?** Mark once (x) for each month.

***This is how you fill in your answers:***

- **If you bike to and from your place of work/study 5 days a week during the whole month, the number of bike rides per weeks will be 10 per week on average.**
- **If, instead, you have half the month off, the number of bike rides will be on average 5 per week during that month.**
- **If you are on holiday the whole month, the number of bike rides will be 0.**
- **If, on average, you make fewer than 1 bike ride per week but in all more than 0, you shall mark the <1 box.**
- **If you bike to and/or from your place of work/study irregularly and on few occasions during the year, and you are uncertain about which months you do so, mark the “*Different alternative*” box.**

| **Month** | Average number of return bike rides per week | | | | | | | | | | | | | | | | | | |
| --- | --- | --- | --- | --- | --- | --- | --- | --- | --- | --- | --- | --- | --- | --- | --- | --- | --- | --- | --- |
|  | 0 | <1 | 1 | 2 | 3 | 4 | 5 | 6 | 7 | 8 | 9 | 10 | 11 | 12 | 13 | 14 | More... | State number: | Don’t know |
| **January** |  |  |  |  |  |  |  |  |  |  |  |  |  |  |  |  |  | ….. |  |
| **February** |  |  |  |  |  |  |  |  |  |  |  |  |  |  |  |  |  | ….. |  |
| **March** |  |  |  |  |  |  |  |  |  |  |  |  |  |  |  |  |  | ….. |  |
| **April** |  |  |  |  |  |  |  |  |  |  |  |  |  |  |  |  |  | ….. |  |
| **May** |  |  |  |  |  |  |  |  |  |  |  |  |  |  |  |  |  | ….. |  |
| **June** |  |  |  |  |  |  |  |  |  |  |  |  |  |  |  |  |  | ….. |  |
| **July** |  |  |  |  |  |  |  |  |  |  |  |  |  |  |  |  |  | ….. |  |
| **August** |  |  |  |  |  |  |  |  |  |  |  |  |  |  |  |  |  | ….. |  |
| **September** |  |  |  |  |  |  |  |  |  |  |  |  |  |  |  |  |  | ….. |  |
| **October** |  |  |  |  |  |  |  |  |  |  |  |  |  |  |  |  |  | ….. |  |
| **November** |  |  |  |  |  |  |  |  |  |  |  |  |  |  |  |  |  | ….. |  |
| **December** |  |  |  |  |  |  |  |  |  |  |  |  |  |  |  |  |  | ….. |  |

**Different alternative ………………….**

1. **How many times do you usually have to stop at red lights during your ride to your place of work/study?** You may count the number of stops you make during a normal day.

    I do not stop
    I stop. State the number of stops:

     1  2  3  4  5  6  7  8  9  10  11  12  13  14  15  16  17  18  19  20  More than 20 times, state number……  Don’t know
2. **Do you become sweaty when you bike to your place of work/study?**

No, never
 Yes, 1-25 % of the times
 Yes, 26-50 % of the times
 Yes, 51-75 % of the times
 Yes, 76-100 % of the times
 Don’t know

1. **Do you usually shower after biking to your place of work/study?**

No, never
 Yes, 1-25 % of the times
 Yes, 26-50 % of the times
 Yes, 51-75 % of the times
 Yes, 76-100 % of the times
 Don’t know

#### Question about your bicycle

1. **What type of bicycle do you use to bike to your place of work/study?**  Bicycle without gears
    Bicycle with gears (2- 4 gears)
    Bicycle with gears (5 gears or more)
    Don’t know

Proceed to the next question if, during the last year, you have on some occasion walked all the way to your place of work/study. If not, proceed to question number 28 on page 8.

| 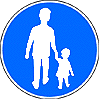 | Questions about your walk to your place of work/study | 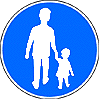 |
| --- | --- | --- |

1. **What is your most frequent route to and from your place of work/study?** Indicate this on the map provided. Follow the attached letter of instruction.

1. **Estimate how long your route is.** State the approximate distance in kilometres. You may use one decimal (e.g. 600 meters = 0.6 km)

   .  km
2. **How much time does the walk usually take from your home to your place of work/study?** Measure the time for the route on an ordinary day when you do not do any errands along the way.   Hours  Minutes
3. **How much time does the walk usually take from your place of work/study to your home?** Measure the time for the route on an ordinary day when you do not do any errands along the way.
    Hours  Minutes
4. **On average, how strenuous do you experience your walk to your place of work/study?** Place one x in each column against a digit on the 6 – 20 scale.

**From your home to your place of work/study**

 6
 7 Very, very light
 8
 9 Very light
 10
 11 Fairly light
 12
 13 Somewhat hard
 14
 15 Hard
 16
 17 Very hard
 18
 19 Very, very hard
 20

**From your place of work/study to your home**

6
 7 Very, very light
 8
 9 Very light
 10
 11 Fairly light
 12
 13 Somewhat hard
 14
 15 Hard
 16
 17 Very hard
 18
 19 Very, very hard
 20

1. **Do you usually walk to and from your place of work/study during the same day?**

     Yes  No  Don’ know

If you have answered No, explain why: ………………………….…………… ………………………………………………………………………………………………

The following is a question, which, combined with the routes you draw on the map, will provide a valuable picture of your physical activity as well as potential health effects of your walking.

1. **How many times do you walk (see instruction below) between your home and your place of work/study, on average, per week during each month?** Tick once (x) for each month.

***This is how you fill in your answers:***

- **If you walk to and from your place of work/study 5 days a week during the whole month the number of walks will be on average 10 per week.**
- **If, instead, you have half the month off, the number of walks will be, on average, 5 per week during that month.**
- **If you are on holiday the whole month, the number of walks will be 0.**
- **If, on average, you walk fewer than 1 time per week but in total more than 0, mark the <1 box.**
- **If you walk to and/or back from your place of work/study irregularly and on a few occasions during the year, and you are uncertain about which months you do so, mark the ”*Different alternative*” box”.**

| **Month** | Number of walks on average per week | | | | | | | | | | | | | | | | | | |
| --- | --- | --- | --- | --- | --- | --- | --- | --- | --- | --- | --- | --- | --- | --- | --- | --- | --- | --- | --- |
|  | 0 | <1 | 1 | 2 | 3 | 4 | 5 | 6 | 7 | 8 | 9 | 10 | 11 | 12 | 13 | 14 | More… | State number: | Don’t know |
| **January** |  |  |  |  |  |  |  |  |  |  |  |  |  |  |  |  |  | …... |  |
| **February** |  |  |  |  |  |  |  |  |  |  |  |  |  |  |  |  |  | …... |  |
| **March** |  |  |  |  |  |  |  |  |  |  |  |  |  |  |  |  |  | …... |  |
| **April** |  |  |  |  |  |  |  |  |  |  |  |  |  |  |  |  |  | …... |  |
| **May** |  |  |  |  |  |  |  |  |  |  |  |  |  |  |  |  |  | …... |  |
| **June** |  |  |  |  |  |  |  |  |  |  |  |  |  |  |  |  |  | …... |  |
| **July** |  |  |  |  |  |  |  |  |  |  |  |  |  |  |  |  |  | …... |  |
| **August** |  |  |  |  |  |  |  |  |  |  |  |  |  |  |  |  |  | …... |  |
| **September** |  |  |  |  |  |  |  |  |  |  |  |  |  |  |  |  |  | …... |  |
| **October** |  |  |  |  |  |  |  |  |  |  |  |  |  |  |  |  |  | …... |  |
| **November** |  |  |  |  |  |  |  |  |  |  |  |  |  |  |  |  |  | …... |  |
| **December** |  |  |  |  |  |  |  |  |  |  |  |  |  |  |  |  |  | …... |  |

Different alternative ………………….

1. **How many times do you usually have to stop at red lights during your walk to your place of work/study?** You may count the number of stops you make during an ordinary day.
    I do not stop
    I stop. State the number of stops:
     1  2  3  4  5  6  7  8  9  10  11  12  13  14  15  16  17  18  19  20  More than 20 times. State the number……….  Don’t know
2. **Do you become sweaty when you walk to your place of work/study?**

No, never
 Yes, 1-25 % of the times
 Yes, 26-50 % of the times
 Yes, 51-75 % of the times
 Yes, 76-100 % of the times
 Don’t know

1. **Do you usually shower after walking to your place of work/study?**

No, never
 Yes, 1-25 of the times
 Yes, 26-50 % of the times
 Yes, 51-75 % of the times
 Yes, 76-100 % of the times
 Don’t know

#### Questions about your physical activity during working/studying hours

1. **How physically strenuous has your daily work or your daily occupation (not spare time) been during the last 12 months?**

    Very easy, predominantly sedentary

    Light physical work, but I do move a good deal (for example light industrial work, shop assistant, teacher)

Physically rather strenuous work (for example cleaner, postman, assistant nurse)

 Physically very strenuous work (heavy manual work, for example bicycle messenger, heavy forestry work, or construction work)

1. **Is it possible for you to do physical exercise during paid working hours?**

    No
    Yes, but I do not take the opportunity to do so
    Yes, and I make use of the opportunity
    Don’t know

If you have answered ”**Yes, and I make use of the opportunity**”, proceed with the next question; if not, proceed to question 32 on page 10.

1. **What type of activity, how often and for how long do you usually do physical exercise/training during paid working hours?** Use an average value if it varies between weeks. You may indicate several activities.
    **Number of occasions Time per** **Activity per week training session**

    Weight training …………… times …….. min

    Fitness training …………… times …….. min

    Ball game …………… times …….. min

    Keep-fit exercise …………… times …….. min
   (e.g. aerobics/Friskis & Svettis)

    Other,
   indicate what:
   ………………………..… …………… times …….. min
   ………………………..… …………… times …….. min ………………………..… …………… times …….. min

1. **On what average level of intensity do you usually exercise/train during paid working hours?** Mark (x) only once. Thus, use an average value for the different activities if you practise different kinds of activities.

6
 7 Very, very light
 8
 9 Very light
 10
 11 Fairly light
 12
 13 Somewhat hard
 14
 15 Hard
 16
 17 Very hard
 18
 19 Very, very hard
 20

**Questions about your physical activity during your spare time, except commuting time**

1. **How much have you generally moved or exerted yourself physically in your *spare time* during the last year? NB! Mark (x) *all* options that apply to you.** Do not include physical activity during commuting, i.e. biking and walking to your place of work/study.

a) I have moved very little.

b) I have moved very little but sometimes taken an odd walk or something like that.

c) I have had ”everyday exercise” in connection with cleaning, climbing stairs, garden work, social dancing, strolling or light bike rides (except walking/biking all the way to the place of work/study), taking the dog for a walk etc.

d) I have, in addition to the activities in c), devoted myself to some light kind of exercise, like strolls (or other activities with similar exertion) *at least once every week*.

e) I have devoted myself to more strenuous exercise, such as quick walks, jogging, swimming, keep-fit exercise or equivalent *at least once every week*.

f) I have regularly devoted myself to *hard training or competition* where the physical exertion has been great, e.g. running and various ball games.

If you have marked the alternatives e) and/or f) proceed with the following question. Others may proceed to question number 35 on the next page.

1. **If you marked the alternatives e) and/or f) in question number 32, what type of activities, how often and for how long do you usually exercise/train?** Use an average value if it varies from one week to another. You may indicate several activities.

**Activity Category Time per** **Number of times No of months** **e) f) occasion per week per year**

Weight training   ……… min ……….... …………
 Fitness training   ……… min ……….... …………
 Ball games   ……… min ……….... …………
 Keep-fit exercise   ……… min ……….... …………
(e.g. aerobics/

Friskis & Svettis)
 Other,
indicate what:
………………   ……… min ……….... …………
………………   ……… min ……….... …………

1. **If you marked the alternatives e) and/or f) of question number 32 and 33, on what average level of exertion do you usually exercise/train?**
   Mark only one option in each column. Thus, use an average value for the various activities if you do several activities within the alternatives e) and f), respectively.  **e) f)**  6
     7 Very, very light
     8
     9 Very light
     10
     11 Fairly light
     12
     13 Somewhat hard
     14
     15 Hard
     16
     17 Very hard
     18
     19 Very, very hard
     20
2. **Are you interested in participating in the next stage of this study?**

   Stage 2. Second part of the survey  Yes  No  Don’t know

   Stage 3. Fitness test and measuring
   energy metabolism during the route.  Yes  No  Don’t know
3. **If you have any comments on this inquiry and its question, you are welcome to write them here and, if necessary, to continue on the back page.**
   ___________________________________________________________________________________________________________________________________________________________________________________________________________________________________________________________________________________________________________________________________________________________________________________________________________________________________________________________________________________________________________________________________________________________________________________________________________

**Many thanks for your help!**
