# Supplementary material for: The heart rate method for estimating oxygen uptake: Analyses of reproducibility using a range of heart rates from cycle commuting
Source: PLoS One. 2019 Jul 24;14(7):e0219741. doi: 10.1371/journal.pone.0219741 (PMC6655643; doi:10.1371/journal.pone.0219741)
Supplement: S3 Methods — The original version in Swedish. (DOC) [file pone.0219741.s003.doc]

# Instruktioner för hur Du fyller i enkäten och kartan

Fyll i med kryss i svarsrutan, så här . Om du kryssar i fel ruta fyller du i hela rutan med färg,

så här  Kryssa sedan i rätt ruta.

Frågorna om din arbetspendling syftar på dina färder under det senaste året till din vanligaste arbets-/studieplats. Tänk på de dagar då du inte utför några ärenden på vägen när du svarar på frågorna.

För att fylla i enkäten behöver du göra fyra enkla saker:

1. mäta hur lång tid det tar att cykla/gå både till och från arbets-/studieplatsen (se frågorna 9, 10, 20, 21),
2. räkna antalet stopp för rödljus som du vanligtvis gör på färdvägen till arbets-/studieplatsen (se frågorna 14 och 25),
3. uppskatta din genomsnittliga ansträngningsnivå under färden (se svarsalternativ i frågorna 11 och 22), samt
4. markera din färdväg mellan bostaden och arbets-/studieplatsen på bifogad karta enligt instruktion nedan.

Uppgifter om din färdväg är av stor betydelse för studien. Om du känner dig osäker på vilka vägar du cyklar/går rekommenderar vi att du cyklar/går sträckan och noterar gatunamnen.

| 1. Fyll i din vanliga färdväg till arbets-/studieplatsen med ett heldraget streck för cykel ___________ och ett streck med kryss för gång  ~~X X X~~ . Markera din bostad med **B** och din arbetsplats med  (se figur 1 och 2). Fyll helst i med en blå eller röd bläck- eller kulspetspenna. Du ska alltså markera för både cykel och gång om du cyklar ibland och går ibland. 2. Om din färdväg hem från arbets-/studieplatsen skiljer sig åt jämfört med vägen dit ber vi dig att dra ett streck med cirklar för hemvägen om du cyklar ~~O O O~~  och ett streck med trianglar om du går  ~~  ~~ (se figur 1 och 2). 3. Om du vanligtvis lämnar barn på förskola/skola ber vi dig markera det med **S** på kartan där förskolan/skolan ligger (se figur 1 och 2). Räkna bort den tid det tar att lämna barnen från färdtiden. 4. Om du går eller cyklar på parkvägar, i tunnlar eller på stigar som inte finns utsatta på kartan ber vi dig rita in den vägen på kartan så noggrant som möjligt. 5. Har du två arbets-/studieplatser ber vi att du fyller i färdvägen till och från den arbets-/studieplats du arbetar mest på. Om du arbetar/studerar lika mycket på två eller flera ställen ber vi dig att bara fylla i färdvägen till en av arbets-/studieplatserna.   Den bifogade kartan är oftast hämtad från telefonkatalogen. Om den inte stämmer kan du antingen komplettera själv genom telefon-katalogens karta i Gula sidorna eller kontakta oss (se nedan) så skickar vi en ny karta. Har du frågor får du gärna ringa tel 08-16 14 53 under dagtid. | Figur 1. Exempel på hur en cykeltur kan markeras på kartan. |
| --- | --- |
|  | Figur 2. Exempel på hur en gångtur kan markeras på kartan. |
