# Supplementary material for: The heart rate method for estimating oxygen uptake: Analyses of reproducibility using a range of heart rates from cycle commuting
Source: PLoS One. 2019 Jul 24;14(7):e0219741. doi: 10.1371/journal.pone.0219741 (PMC6655643; doi:10.1371/journal.pone.0219741)
Supplement: S5 Methods — The original version in Swedish. (DOC) [file pone.0219741.s005.doc]

Information om de fysiologiska studierna av fysiskt aktiv arbetspendling

# Syfte

Det övergripande syftet med studien är att belysa hur det fysiska arbetet, uttryckt i fysiologiska termer, ter sig vid promenad mellan bostad och arbets-/studieplatsen. Med detta menar vi vilken syreupptagning och energiomsättning som arbetet genomförs med. Dessa nivåer vill vi kunna relatera till din maximala syreupptagning och hjärtfrekvens för att bättre förstå hur arbetet kan påverka din hälsa och välbefinnande. Under vila mäter vi också hur din hjärtfrekvens varierar samt blodtrycket, två mätningar som kan ge upplysningar om din hälsa.

För det ändamålet behöver vi genomföra studier både på laboratoriet på GIH och under din cykeltur mellan bostad och arbete.

För mätningarna under din promenad kommer vi att använda en mobil utrustning (se Figur 1) som under flera år har utvecklats i samarbete med GIH och Riksidrottsförbundets elitidrottscentrum på Bosön, och nu är färdig för användning. Nedan beskrivs de olika försöken mer i detalj.


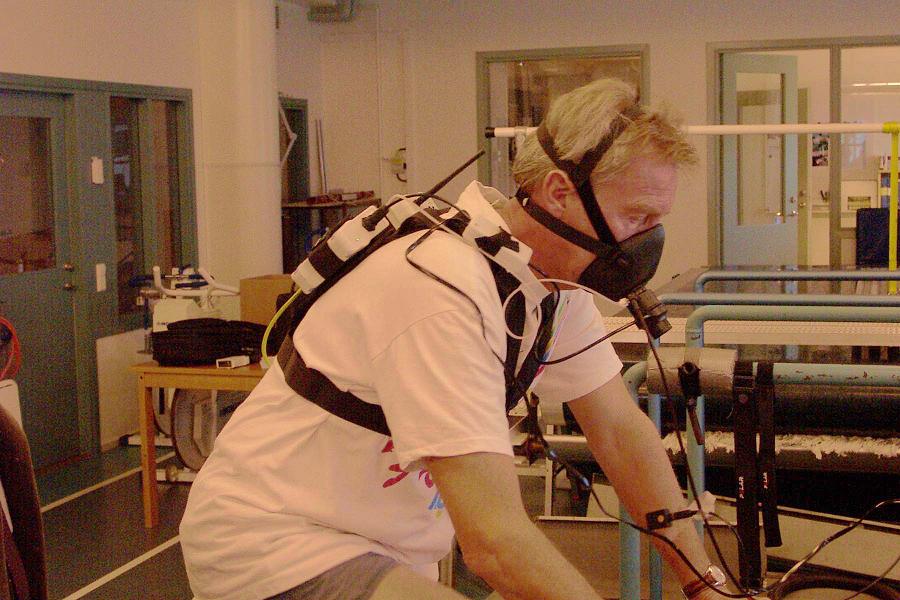


Figur 1. Mobilt system för uppkopplade meta-bola mätningar av andetag för andetag med Jæger Oxycon Mobile.

# Försöken på GIH:s laboratorium

Vid testerna på GIH mäts först blodtrycket och din hjärtfrekvens samt dess variation i vila. Därefter mäts din syreupptagning och hjärtfrekvens vid olika stegrade arbetsbelastningar. De olika tillfällena behövs för att du ska vänja dig vid testen och att vi ska kunna mäta syreupptagningen både under cykelarbete och förhoppningsvis även på ett s.k. löpande band.

Tillfälle 1 och 2

# *Vilotest*

Vi inleder med hjärtfrekvensregistrering under vila och sedan vidtar blodtrycksmätning.

*Cykeltest*

Du får sedan cykla på en motionscykel vid tre olika, lättare arbetsbelastningar. Därefter stegras arbetsbelastningarna varje minut tills du når din maximala syreupptagning. Under arbetena samlar vi in din utandningsluft genom att du andas i en mask som täcker näsan och munnen (se bilden ovan). Genom en pulsklocka mäter vi samtidigt hur hjärtat arbetar. Efter varje arbete anger du hur ansträngande arbetet upplevdes med hjälp av en särskild skattningsskala. Första tillfället tar c:a 1 tim och 30 min, nästa tillfälle tar c:a 1 tim och 15 min.

Tillfälle 3

Löptest på löpband

Efter en uppvärmning i form av promenad och lättare löpning genomförs en maximal test. Det innebär att du springer på löpband vars lutning höjs några grader varje minut tills du springer ditt absoluta maximum. Löptestet tar c:a 45 minuter. Om du av någon anledning inte kan eller vill utföra löptestet, t.ex. en knäskada, får du avstå från det.

**Fälttest**

Tillfälle 4

Vi kommer då till dig i din normala miljö och har med oss den mobila syreupptagnings- utrustningen. Du går sedan mellan bostaden och arbets-/studieplatsen med den utrustningen och en pulsklocka på dig. Dessutom har du en stegräknare och en s.k. accelerometer på dig. Den senare registrerar din kropps antal rörelser i olika riktningar med tiden.

En testledare sätter på utrustningen vid starten och tar hand om den när du kommit fram. Då tar vi dessutom ett mycket litet blodprov i en fingerspets för att mäta blodets nivå av mjölksyra.

###### **Standardiseringskrav**

Följande standardiseringskrav för alla de fyra testtillfällena ber vi att du följer dessa punkter:

- utför eventuell hård träning mer än 24 timmar före testtillfället
- äter eventuell lätt måltid mer än 1 timme före testtillfället
- äter eventuell tyngre måltid mer än 3 timmar före testtillfället
- inte röker/snusar mindre än 1 timme före respektive testtillfället
- ej har feber, infektion eller förkylning, utan då ställs istället mätningen in

###### **Klädsel**

###### Vid tillfälle 1-3 ska du ha lätt klädsel såsom: T-shirt, kortbyxor, träningsskor. Vid tillfälle 4 ska du ha din normala klädsel under den fysiskt aktiva arbetspendlingen.

###### **Arvode**

250:- per tillfälle. Arvodet är skattepliktigt.

##### **Deltagande- och hälsodeklaration**

#####

##### Svarsformulär

Namn och deltagande

Namn:

Jag har tagit del av ovanstående information om försöken och:

vill delta i försöken  Om ja, vänligen fortsätt besvara nedanstående hälsodeklaration

vill ej delta i försöken  Om nej, stort tack för ditt svar! Nyttja svarskuvertet och lägg det i brevlådan.

# *Hälsodeklaration*

**Medicinering och hälsostatus**

Använder Du medicin regelbundet?

🞏 Jag använder inga mediciner

🞏 Jag använder följande mediciner mot följande sjukdomar:

Har du upplevt hjärtklappning, smärta i bröstet eller onormalt tung andning vid fysisk ansträngning?

🞏 Ja 🞏 Nej

Om Ja, ange när:

Har du högt blodtryck?

🞏 Ja 🞏 Nej

Om Ja, vet du vilka dina värden är:

Har du undvikit eller avbrutit träning den senaste tiden p.g.a. skada eller av hälsoskäl?

🞏 Ja 🞏 Nej

Om Ja, ange orsak:

Förutsättningar för deltagande i testen och hälsodeklaration

Undertecknad testperson har erhållit information om testerna samt deltager frivilligt och på egen risk i dessa, med vetskap om möjligheten till avbrytande av test när som helst och utan krav på förklaring till detta.

Undertecknad testperson uppfattar sig som fullt frisk och ser inga medicinska hinder för deltagande i testerna.

Ort:…………………………………… den / 2006

Testpersonens namnteckning

Testpersonens personnummer

**Stort tack för ditt svar!**

Nyttja svarskuvertet och lägg det i brevlådan**.**
