# Supplementary material for: The heart rate method for estimating oxygen uptake: Analyses of reproducibility using a range of heart rates from cycle commuting
Source: PLoS One. 2019 Jul 24;14(7):e0219741. doi: 10.1371/journal.pone.0219741 (PMC6655643; doi:10.1371/journal.pone.0219741)
Supplement: S6 Methods — The original version in Swedish, translated into English. (DOC) [file pone.0219741.s006.doc]

**Translated from Swedish.**

**Information about the physiological studies of active commuting**

**between home and work or study place**

# Purpose

The overall purpose of the study is to illuminate how physical exercise, expressed in physiological terms, presents itself during bike rides between one’s home and place of work or study. By this we mean the oxygen uptake and the metabolism with which the cycling is carried out. We want to relate these levels to your maximum oxygen uptake and your heart rate in order to understand better how cycling may impact your health and wellbeing. During rest we also measure variations in your heart rate and blood pressure, two measurements which can provide information about your health.

For this purpose we need to perform studies in the laboratory at the Swedish School of Sport and Health Sciences, GIH, as well as during your bike ride between home and place of work or study.


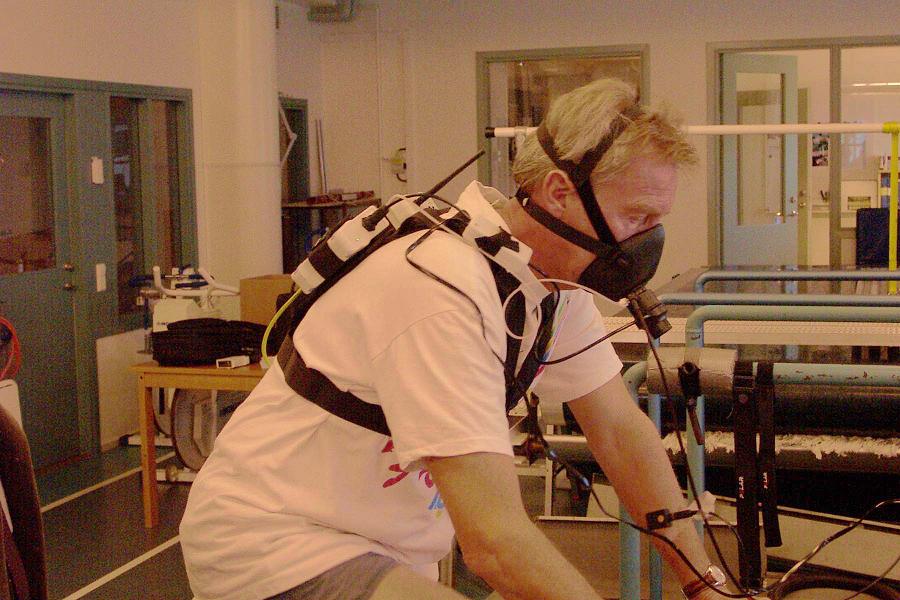
For measuring during your bike ride we will use a mobile equipment (see Figure 1) which has been developed in collaboration with GIH and the Elite Sports Centre of the Swedish Sports Confederation at Bosön, Lidingö, during several years and is now ready to be applied. See below for a more detailed description of the various tests.

Figure 1. Mobile system for on-line breath by breath metabolic measurements using the Jæger Oxycon Mobile

# The laboratory tests at the GIH

For the tests at the GIH, first of all your blood pressure and your heart rate will be measured, as well its variation during rest. After that your oxygen uptake and your heart rate will be measured on various increased levels of work rate. Several occasions are necessary to make you familiar with the tests and for us to measure the oxygen uptake both during cycling and, hopefully, also on a so called treadmill.

Occasions No 1 and 2

# *Test during rest*

We will start with heart rate registration during rest, followed by blood pressure measurement.

*Cycling test*

Then you will ride on an ergometer cycle at three different, light work rates. Subsequently, the work rates will be increased each minute until you reach your maximum oxygen uptake. During the sessions we will collect your exhaled air through the mask, covering your nose and mouth, in which you will breathe (picture above). Simultaneously, a heart rate monitor watch will measure how your heart is working. After every session you will indicate on a special rating scale how strenuous you found the exercise. The first session will take approximately 1 hour and 30 minutes. The next occasion will take approximately 1 hour and 15 minutes.

Occasion No 3

Running test on a treadmill

After warming up with a walk and a light run, a maximum test is performed. This means that you will run on a treadmill with a slope that will be increased by some degrees every minute until you will be running at your absolute maximum. The running test will take approximately 45 minutes. If, for some reason, you cannot or do not want to perform the running test, e.g. a knee injury, you may refrain.

**Field test**

Occasion No 4

We will come to you in your everyday context, bringing the mobile oxygen uptake equipment. Then you will cycle between your home and your place of work/study wearing this equipment and a heart rate monitor. You will also have a pedometer and a so called accelerometer attached to your leg. They will register your leg movements with time passing.

A test leader will put on the equipment at the start and take care of it when you arrive. Then we will also take a very small blood sample in a fingertip to measure the level of lactic acid in your blood.

###### **Standardisation requirements**

We ask you to abide by the following standardization requirements on all test occasions:

– hard training should not be performed less than 24 hours before the test occasion

– any possible light meal should be taken more than 1 hour before the test occasion

- any possible more substantial meal should be taken more than 3 hours before the test occasion
- do not smoke or take snuff less than 1 hour before the test occasion
- the test should not be undertaken if you have fever, infection or cold

###### **Clothing**

###### On occasion No 1-3 you should wear light clothes such as: T-shirt, shorts and training shoes. On occasion No 4 you should wear the clothes you normally wear while actively commuting to your work or study place.

###### **Remuneration**

250:- per occasion. The remuneration is taxable.

# Participation and Health Declaration

##### Reply form

Name and participation particulars

Name:

I have considered the information above and I:

| want to participate in the tests | If yes, please continue by answering the  health declaration below |
| --- | --- |
| do not want to participate in the tests | If no, many thanks for answering! Use the  return envelope and put it in a mailbox. |

# *Health declaration*

**Medication and health status**

Do you regularly use any medication?

🞏 I do not use any medication

🞏 I use the following medication against these disorders:

Have you experienced palpitation of the heart, chest pains or abnormally heavy breathing in connection with physical strain?

🞏 Yes 🞏 No

If Yes, indicate when:

Do you have high blood pressure?

🞏 Yes 🞏 No

If Yes, do you know your readings? :

Have you avoided or interrupted physical training lately because of an injury or for health reasons?

🞏 Yes 🞏 No

If Yes, indicate the reason:

Conditions for participating in the test, and a health declaration

I, the undersigned test person, have received information about the tests and will participate in these voluntarily and at my own risk, being aware of the possibility to break off a test at any time and without any demand for explanation.

I, the undersigned test person, consider myself in perfect health and can see no medical obstacle for participating in the tests.

Place and date:…………………… on the .............. 2006

Test person’s signature

Test person’s civic registration number

**Thank you very much for answering!**

Use the reply envelope and put it into a mailbox**.**
