# Supplementary material for: The heart rate method for estimating oxygen uptake: Analyses of reproducibility using a range of heart rates from cycle commuting
Source: PLoS One. 2019 Jul 24;14(7):e0219741. doi: 10.1371/journal.pone.0219741 (PMC6655643; doi:10.1371/journal.pone.0219741)
Supplement: S7 Methods — The original version in Swedish. (DOC) [file pone.0219741.s007.doc]

**Enkät nr 2**

**om fysisk aktivitet**

**vid arbetspendling**

# Enkät till dig som går eller cyklar till arbets-/studieplatsen

#### Instruktioner för hur du fyller i enkäten

Fyll i med kryss i svarsrutan, så här . Om du kryssar i fel ruta fyller du i hela rutan med färg, så här  Kryssa sedan i rätt ruta.

#### Frågor om restider och förhållande till andra färdmedel

1. **När har du vanligtvis gått/cyklat hemifrån till din arbets-/studieplats de senaste två veckorna?**

Mellan 5.00 och 6.00

Mellan 6.01 och 7.00

Mellan 7.01 och 8.00

Mellan 8.01 och 9.00

Mellan 9.01 och 10.00

Annan tid ……….

1. **När har du vanligtvis gått/cyklat iväg från din arbets-/studieplats de senaste två veckorna?**

Mellan 14.00 och 15.00

Mellan 15.01 och 16.00

Mellan 16.01 och 17.00

Mellan 17.01 och 18.00

Mellan 18.01 och 19.00

Mellan 19.01 och 20.00

Annan tid ……….

1. **Hur lång tid skulle din färd till arbets-/studieplatsen ta om du istället valde den snabbaste vägen med kollektivtrafik motsvarande tid på dygnet?**

**………** timmar **………** minuter Vet ej

1. Hur lång tid skulle din färd till arbets-/studieplatsen ta om du istället valde den snabbaste vägen med bil motsvarande tid på dygnet?

**………** timmar **………** minuter  Vet ej

**Nu kommer en fråga till dig som går:**

# Vilket transportsätt skulle du välja om du inte kunde gå hela vägen till din arbets-/studieplats?

Bil
 Bil som passagerare
 Kollektivtrafik
 Moped
 MC
 Cykel
 Kombination av flera transportsätt: Ange vilka……………………..

Annat färdsätt: …………………….
 Vet ej

**Nu kommer en fråga till dig som cyklar:**

# Vilket transportsätt skulle du välja om du inte kunde cykla hela vägen till din arbets-/studieplats?

Bil
 Bil som passagerare
 Kollektivtrafik
 Moped
 MC
 Gång
 Kombination av flera transportsätt: Ange vilka……………………..
 Annat färdsätt: …………………….
 Vet ej

1. **Har du körkort för personbil?**

Ja  Nej  Vet ej

1. **Har du vanligtvis tillgång till en personbil?**

Nej

Ja, min egen
 Ja, en bil i hushållet
 Ja, genom en bilpool
 Ja, jag har möjlighet att åka med i någon annans bil
 Ja, flera bilar.
 Vet ej

1. **Har du tillgång till en parkeringsplats för bil på din arbets-/studieplats?**

Nej

Ja, gratis

Ja, avgiftsbelagd

Vet ej

1. **Hur är möjligheterna att parkera nära din bostad?**

Jag har egen parkeringsplats

Mycket bra

Ganska bra

Ganska dåliga

Mycket dåliga

Vet ej

1. **Är det gratis/avgiftsbelagt att parkera vid din bostad?**

Är det t.ex. gratis på natten och helgen och avgiftsbelagt på dagen kryssar du i alternativet ”avgiftsbelagt”.

Gratis

Avgiftsbelagt någon gång under veckan

Vet ej

1. **Har du haft tillgång till ett SL-kort de senaste två veckorna?**
   Med SL-kort menar vi årskort, säsongskort/terminskort eller månadskort.

Nej

Ja, eget

Ja, lånat

Vet ej

## Bakgrundsfrågor

1. **Var bodde du huvudsakligen under din barn- och ungdomstid?**

Storstad inklusive förorter (t.ex. Stockholm, Göteborg, Malmö)
 Annan stad
 Mindre samhälle
 Landsbygd
 Vet ej

1. **Hur tog du dig vanligtvis till skolan när du var ca 12 år?**

Åkte skolbuss/skoltaxi större delen av vägen

Blev skjutsad i bil

Cyklade en kort väg (mindre eller lika med 1 km) till skolan

Cyklade en lång väg (mer än 1 km) till skolan

Gick en kort (mindre eller lika med 1 km) väg till skolan

Gick en lång väg (mer än 1 km) till skolan

Annat färdsätt

Minns ej

1. **Ägnade du dig åt idrott på fritiden under din barn- och/eller ungdomstid?**Sätt ett eller flera kryss!

Nej

Ja, i en idrottsförening och tävlade
 Ja, i en idrottsförening men tävlade ej
 Ja, tillsammans med kamrater och/eller på egen hand
 Ja, tillsammans med familjen
 Vet ej

1. **Ägnade du dig åt friluftsliv på fritiden under din barn- och/eller ungdomstid?**Sätt ett eller flera kryss!

Nej

Ja, friluftsverksamhet i en förening
 Ja, tillsammans med kamrater och/eller på egen hand
 Ja, tillsammans med familjen
 Vet ej

1. **Var du medlem i någon miljöförening under din barn och/eller ungdomstid?**

Ja  Nej  Vet ej

1. **Vilket var ditt senaste betyg i ämnet gymnastik/idrott & hälsa?**

Ange betyg: *……………*  Vet ej

## Allmänna frågor

1. **Hur mycket har du i genomsnittlig inkomst per månad före skatt?**

Med inkomst menar vi lön, pension, ersättning, bidrag, studiemedel, inkomst av eget företag.

Har ingen inkomst alls

0-10 000 kr

10 001 - 15 000 kr

15 001 - 20 000 kr

20 001 - 25 000 kr

25 001 - 30 000 kr

30 001 - 35 000 kr

35 001 kr eller mer

1. **Vilken är den högsta utbildning du har genomgått?**Sätt bara ett kryss!

Folkskola
 Grundskola
 Grundsärskola
 Realskola
 Yrkesskola eller motsvarande

Fackskola, 2-årig gymnasieskola
 3-4 årig gymnasieskola
 Folkhögskola
 Universitet eller högskola
 Annan utbildning, vilken? ………………..
 Vet ej

Nu följer två frågor riktade till dig som arbetar. Om du inte arbetar utan t.ex. studerar gå direkt till fråga 23.

1. **Vilken är din huvudsakliga arbetsgivare?**

Stort privat företag

Litet privat företag

Ideell organisation/förening
 Statligt verk eller myndighet
 Kommun
 Landsting

Vet ej

1. **I vilken bransch arbetar du?**Välj den bransch som passar bäst.

Jordbruk, jakt och skogsbruk

Tillverkning

El-, gas, värme- och vattenförsörjning

Byggverksamhet

Partihandel och detaljhandel

Hotell- och restaurangverksamhet

Transport, magasinering

Post och telekommunikation

Finansiell verksamhet t.ex. bank och försäkring

Fastighets- och uthyrningsverksamhet

Företagstjänster ex. dataverksamhet

Forskning och utveckling

Offentlig förvaltning och försvar

Utbildning

Hälso- och sjukvård, sociala tjänster, veterinärverksamhet

Andra samhälleliga och personliga tjänster ex. kultur, religion, rekreation, sport.

Övrigt, ange bransch ……………….

1. **I vilken typ av bostad bor du?**

Flerfamiljshus (hyr)

Flerfamiljshus (bostadsrätt)

Radhus/villa/enfamiljshus (hyr)

Radhus/villa/enfamiljshus (äger själv, bostadsrätt)

Vet ej

1. **Delar du bostad med någon?**

D.v.s. vem/vilka bor du tillsammans med under större delen av veckan. Du kan ange flera alternativ!

Nej

Ja, Föräldrar/syskon

Ja, Make/maka/sambo/partner

Ja, Andra vuxna

Ja, Barn, hur många:…….st 🡪 Hur gamla är de?

Ange antal vid respektive åldersgrupp:

0-6 år ….. st

7-12 år ….. st

13-17 år ….. st

18 år eller äldre….. st

1. **Har du eller dina föräldrar invandrat till Sverige?**

Du kan ange flera alternativ

Nej

Ja, jag själv från………………………….…

Ja, mamma från…………………..…….…..

Ja, pappa från ………………………………

Annat alternativ ……………………………

Vet ej

1. **Är du medlem i någon idrottsförening?**

Nej

Ja, som ledare

Ja, som aktiv, även i tävlingsverksamhet

Ja, som aktiv motionär

Ja, som passiv medlem

Vet ej

1. **Är du medlem i någon friluftsförening?**

Nej

Ja, som aktiv medlem

Ja, som passiv medlem

Vet ej

1. **Är du medlem i någon miljöförening?**

Nej

Ja, som aktiv medlem

Ja, som passiv medlem

Vet ej

#### Frågor om ditt hälsotillstånd och din livsstil

1. **Hur är ditt allmänna fysiska hälsotillstånd?**

Mycket dåligt

Dåligt

Inte särskilt bra

Godtagbart

Ganska bra

Bra

Mycket bra

Vet ej

1. **Hur är ditt allmänna psykiska hälsotillstånd?**

Mycket dåligt

Dåligt

Inte särskilt bra

Godtagbart

Ganska bra

Bra

Mycket bra

Vet ej

1. **Har du varit sjukskriven de senaste 12 månaderna?**

Räkna inte med vård av barn.

Nej  Ja, totalt ca……… dagar  Vet ej

1. **Röker du?**

Nej

Ja, varje dag minst en gång

Ja, ibland

Vet ej

1. **Har du något medicinskt/fysiskt handikapp och/eller astma, allergiska besvär som hindrar fysisk aktivitet?**

Nej  Ja, nämligen……………………………  Vet ej

1. **Är du intresserad av att delta i enkätens tredje steg?**

    Ja  Nej  Vet ej

#### Fråga till dig som flyttat eller bytt arbets-/studieplats

1. Om du har en annan färdväg än den du angav på kartan i september 2004 och fortfarande går eller cyklar till din arbets-/studieplats ber vi dig att ange din nya bostadsadress samt ny adress till arbets-/studieplatsen.

Ny bostad: Ny arbets-/studieplats:

# Gatuadress: …………………………. ……………………………

# Postnummer: …………………….… ……………………………

**Postort**: ……………………………..... ……………………………

### Frågor om din färdväg

Nu kommer ett antal frågor om hur du uppfattar miljön som du har gått och/eller cyklat i på väg till arbets-/studieplatsen under de senaste två veckorna. Ange din helhetsupplevelse under dessa veckor. Vi ber dig skilja på upplevelser när färdvägen går i innerstadsmiljö respektive när färden går i ytterstadsmiljö (se figur 1 nedan). Ange upplevelser i innerstadsmiljö på rad 1 och i ytterstadsmiljö på rad 2, se exempelrutan längst ned på sidan.

**Lill-Jans skogen**

**Norra ytterstaden**

**KTH**

##### Gärdet

**Brunnsviken**

**Solna**

**Östermalm**

**Kungsholmen**

**Årsta**

**Södermalm**

**Liljeholmen**

**Stora Essingen**

**Bromma**

**Vasastan**

**City**

##### Nacka

**Innerstaden**

**Södra ytterstaden**

Figur 1. Med innerstaden menar vi området innanför den

streckade linjen och med ytterstaden menar vi resten av

Stockholms län. Ex. Gamla stan = innerstad, Täby och Huddinge = ytterstad.

# Exempel på hur frågorna kan fyllas i.

# Ringa in den siffra som bäst stämmer med din upplevelse. Om du ringar in fel eller ändrar dig ber vi dig att kryssa över det felaktiga och ringa in det rätta alternativet. Se exemplet.

**Innerstaden:** Lite 1---2---3---4---5---6---7---8---9---10---11---12---13---14---15 Mycket

varken mycket

eller lite

**Ytterstaden:** Lite 1---2---3---4---5---6---7---8---9---10---11---12---13---14---15 Mycket

varken mycket

eller lite

Om du cyklar/går i båda miljöerna fyller du i båda raderna. Om du först cyklar i södra ytterstaden och sedan passerar innerstaden och avslutar resan i norra ytterstaden anger du ett medelvärde för båda ytterstadsfärderna.

### Frågor om de miljöer du har cyklat i

Alla frågor nedan gäller din helhetsupplevelse av din färdväg som cyklist till arbets-/studieplatsen. Ringa in den siffra som bäst stämmer med din upplevelse.

# Hur upplever du miljön som helhet under färdvägen?

**Innerstaden**: Mycket 1---2---3---4---5---6---7---8---9---10---11---12---13---14---15 Mycket

dålig bra

varken dålig

eller bra

Ytterstaden: Mycket 1---2---3---4---5---6---7---8---9---10---11---12---13---14---15 Mycket

dålig bra

varken dålig

eller bra

1. **Tycker du att miljön som du cyklar i som helhet stimulerar till/motverkar din**

**arbetspendling?**

**Innerstaden**: Motverkar 1---2---3---4---5---6---7---8---9---10---11---12---13---14---15 Stimulerar

mycket mycket

varken motverkar

eller stimulerar

**Ytterstaden**: Motverkar 1---2---3---4---5---6---7---8---9---10---11---12---13---14---15 Stimulerar

mycket mycket

varken motverkar

eller stimulerar

1. **Hur uppfattar du avgasnivåerna under din färdväg?**

**Innerstaden**: Mycket 1---2---3---4---5---6---7---8---9---10---11---12---13---14---15 Mycket

låga höga

varken låga

eller höga

**Ytterstaden**: Mycket 1---2---3---4---5---6---7---8---9---10---11---12---13---14---15 Mycket

låga höga

varken låga

eller höga

1. **Hur uppfattar du bullernivåerna under din färdväg?**

**Innerstaden**: Mycket 1---2---3---4---5---6---7---8---9---10---11---12---13---14---15 Mycket

låga höga

varken låga

eller höga

**Ytterstaden**: Mycket 1---2---3---4---5---6---7---8---9---10---11---12---13---14---15 Mycket

låga höga

varken låga

eller höga

1. **Hur uppfattar du flödet av motorfordon (antalet bilar) längst din färdväg?**

**Innerstaden**: Mycket 1---2---3---4---5---6---7---8---9---10---11---12---13---14---15 Mycket

lågt högt

varken lågt

eller högt

**Ytterstaden**: Mycket 1---2---3---4---5---6---7---8---9---10---11---12---13---14---15 Mycket

lågt högt

varken lågt

eller högt

1. **Hur uppfattar du hastigheterna på motorfordon (taxi, lastbil, personbil, buss)**

**under din färdväg?**

**Innerstaden**: Mycket 1---2---3---4---5---6---7---8---9---10---11---12---13---14---15 Mycket

låga höga

varken låga

eller höga

**Ytterstaden**: Mycket 1---2---3---4---5---6---7---8---9---10---11---12---13---14---15 Mycket

låga höga

varken låga

eller höga

1. **Hur du uppfattar andra cyklisters hastigheter under din färdväg?**

**Innerstaden**: Mycket 1---2---3---4---5---6---7---8---9---10---11---12---13---14---15 Mycket

låga höga

varken låga

eller höga

**Ytterstaden**: Mycket 1---2---3---4---5---6---7---8---9---10---11---12---13---14---15 Mycket

låga höga

varken låga

eller höga

1. Hur uppfattar du som cyklist trängselnivåerna i blandtrafik, orsakad av alla

sorters fordon, under din färdväg?

**Innerstaden**: Mycket 1---2---3---4---5---6---7---8---9---10---11---12---13---14---15 Mycket

låga höga

varken låga

eller höga

**Ytterstaden**: Mycket 1---2---3---4---5---6---7---8---9---10---11---12---13---14---15 Mycket

låga höga

varken låga

eller höga

1. **Hur uppfattar du trängselnivåerna orsakad av antalet cyklister på**

**cykelbana/cykelfält under din färdväg?**

**Innerstaden**: Mycket 1---2---3---4---5---6---7---8---9---10---11---12---13---14---15 Mycket

låga höga

varken låga

eller höga

**Ytterstaden**: Mycket 1---2---3---4---5---6---7---8---9---10---11---12---13---14---15 Mycket

låga höga

varken låga

eller höga

1. **Hur uppfattar du förekomsten av konflikter mellan dig som cyklist och andra trafikanter (inklusive fotgängare) under din färdväg?**

**Innerstaden**: Mycket 1---2---3---4---5---6---7---8---9---10---11---12---13---14---15 Mycket

låg hög

varken låg

eller hög

**Ytterstaden**: Mycket 1---2---3---4---5---6---7---8---9---10---11---12---13---14---15 Mycket

låg hög

varken låg

eller hög

1. Ungefär hur stor del av din färdväg består av cykelbana/cykelfält/cykelväg

separerad från bilar? Ringa in ungefärlig andel.

**Innerstaden**: 0 % ---- 10 ---- 20 ---- 30 ---- 40 ---- 50 ---- 60 ---- 70 ---- 80 ---- 90 ---- 100 %

**Ytterstaden**: 0 % ---- 10 ---- 20 ---- 30 ---- 40 ---- 50 ---- 60 ---- 70---- 80 ---- 90 ---- 100 %

1. **Hur otrygg/trygg känner du dig i trafiken som cyklist under din färd?**

**Innerstaden**: Mycket 1---2---3---4---5---6---7---8---9---10---11---12---13---14---15 Mycket

otrygg trygg

varken otrygg

eller trygg

**Ytterstaden**: Mycket 1---2---3---4---5---6---7---8---9---10---11---12---13---14---15 Mycket

otrygg trygg

varken otrygg eller trygg

1. **Hur uppfattar du tillgången på grönska (naturområden, parker, planteringar,**

**träd) längs med färdvägen?**

**Innerstaden**: Mycket 1---2---3---4---5---6---7---8---9---10---11---12---13---14---15 Mycket

låg hög

varken låg

eller hög

**Ytterstaden**: Mycket 1---2---3---4---5---6---7---8---9---10---11---12---13---14---15 Mycket

låg hög

varken låg

eller hög

# Hur fula/vackra uppfattar du att omgivningarna kring din färdväg är?

**Innerstaden**: Mycket 1---2---3---4---5---6---7---8---9---10---11---12---13---14---15 Mycket

fula vackra

varken fula

eller vackra

**Ytterstaden**: Mycket 1---2---3---4---5---6---7---8---9---10---11---12---13---14---15 Mycket

fula vackra

varken fula eller vackra

# Hur mycket upplever du att din cykeltur försvåras av färdvägens dragning?

# T.ex. dragning med många tvära svängar, omvägar, riktningsförändringar, sidbyten osv.

#

**Innerstaden**: Väldigt 1---2---3---4---5---6---7---8---9---10---11---12---13---14---15 Väldigt

lite mycket

varken lite

eller mycket

**Ytterstaden**: Väldigt 1---2---3---4---5---6---7---8---9---10---11---12---13---14---15 Väldigt

lite mycket

varken lite

eller mycket

# Hur mycket upplever du att din cykeltur försvåras av färdvägens backighet? Utgå från färdvägen till och från arbets-/studieplatsen.

**Innerstaden**: Väldigt 1---2---3---4---5---6---7---8---9---10---11---12---13---14---15 Väldigt

lite mycket

varken lite

eller mycket

**Ytterstaden**: Väldigt 1---2---3---4---5---6---7---8---9---10---11---12---13---14---15 Väldigt

lite mycket

varken lite

eller mycket

# Hur mycket upplever du att din framkomlighet försämras av antalet rödljus under din färd till arbets-/studieplatsen?

**Innerstaden**: Väldigt 1---2---3---4---5---6---7---8---9---10---11---12---13---14---15 Väldigt

lite mycket

varken lite

eller mycket

**Ytterstaden**: Väldigt 1---2---3---4---5---6---7---8---9---10---11---12---13---14---15 Väldigt

lite mycket

varken lite

eller mycket

# Hur kort/lång upplever du att din färdväg är?

**Innerstaden**: Väldigt 1---2---3---4---5---6---7---8---9---10---11---12---13---14---15 Väldigt

kort lång

varken kort

eller lång

**Ytterstaden**: Väldigt 1---2---3---4---5---6---7---8---9---10---11---12---13---14---15 Väldigt

kort lång

varken kort

eller lång

### Frågor om de miljöer du har gått i

Alla frågor nedan gäller din helhetsupplevelse av din färdväg som fotgängare till arbets-/studieplatsen. Ringa in den siffra som bäst stämmer med din upplevelse.

# Hur upplever du miljön som helhet under färdvägen?

# Ringa in den siffra som bäst stämmer med din upplevelse.

**Innerstaden**: Mycket 1---2---3---4---5---6---7---8---9---10---11---12---13---14---15 Mycket

dålig bra

varken dålig

eller bra

Ytterstaden: Mycket 1---2---3---4---5---6---7---8---9---10---11---12---13---14---15 Mycket

dålig bra

varken dålig

eller bra

1. **Tycker du att miljön som du går i som helhet stimulerar till/motverkar din**

**arbetspendling?**

**Innerstaden**: Motverkar 1---2---3---4---5---6---7---8---9---10---11---12---13---14---15 Stimulerar

mycket mycket

varken motverkar

eller stimulerar

**Ytterstaden**: Motverkar 1---2---3---4---5---6---7---8---9---10---11---12---13---14---15 Stimulerar

mycket mycket

varken motverkar

eller stimulerar

1. **Hur uppfattar du avgasnivåerna under din färdväg?**

**Innerstaden**: Mycket 1---2---3---4---5---6---7---8---9---10---11---12---13---14---15 Mycket

låga höga

varken låga

eller höga

**Ytterstaden**: Mycket 1---2---3---4---5---6---7---8---9---10---11---12---13---14---15 Mycket

låga höga

varken låga

eller höga

1. **Hur uppfattar du bullernivåerna under din färdväg?**

**Innerstaden**: Mycket 1---2---3---4---5---6---7---8---9---10---11---12---13---14---15 Mycket

låga höga

varken låga

eller höga

**Ytterstaden**: Mycket 1---2---3---4---5---6---7---8---9---10---11---12---13---14---15 Mycket

låga höga

varken låga

eller höga

1. **Hur uppfattar du flödet av motorfordon (antalet bilar) längs din färdväg?**

**Innerstaden**: Mycket 1---2---3---4---5---6---7---8---9---10---11---12---13---14---15 Mycket

lågt högt

varken lågt

eller högt

**Ytterstaden**: Mycket 1---2---3---4---5---6---7---8---9---10---11---12---13---14---15 Mycket

lågt högt

varken lågt

eller högt

1. **Hur uppfattar du hastigheterna på motorfordon (taxi, lastbil, personbil, buss) i**

**gaturummet under din färdväg?**

**Innerstaden**: Mycket 1---2---3---4---5---6---7---8---9---10---11---12---13---14---15 Mycket

låga höga

varken låga

eller höga

**Ytterstaden**: Mycket 1---2---3---4---5---6---7---8---9---10---11---12---13---14---15 Mycket

låga höga

varken låga

eller höga

1. **Hur uppfattar du trängselnivåerna bland fotgängare på gångbanan under din**

**färdväg?**

**Innerstaden**: Mycket 1---2---3---4---5---6---7---8---9---10---11---12---13---14---15 Mycket

låga höga

varken låga

eller höga

**Ytterstaden**: Mycket 1---2---3---4---5---6---7---8---9---10---11---12---13---14---15 Mycket låga höga

varken låga

eller höga

1. **Hur uppfattar du förekomsten av konflikter mellan olika trafikanter (inklusive**

**fotgängare) under din färdväg?**

**Innerstaden**: Mycket 1---2---3---4---5---6---7---8---9---10---11---12---13---14---15 Mycket

låg hög

varken låg

eller hög

**Ytterstaden**: Mycket 1---2---3---4---5---6---7---8---9---10---11---12---13---14---15 Mycket

låg hög

varken låg

eller hög

1. **Hur otrygg/trygg känner du dig i trafiken som fotgängare under din färd?**

**Innerstaden**: Mycket 1---2---3---4---5---6---7---8---9---10---11---12---13---14---15 Mycket

otrygg trygg

varken otrygg

eller trygg

**Ytterstaden**: Mycket 1---2---3---4---5---6---7---8---9---10---11---12---13---14---15 Mycket

otrygg trygg

varken otrygg

eller trygg

#

# Hur uppfattar du tillgången på grönska (naturområden, parker, planteringar,

# träd) längs med färdvägen?

**Innerstaden**: Mycket 1---2---3---4---5---6---7---8---9---10---11---12---13---14---15 Mycket

låg hög

varken låg

eller hög

**Ytterstaden**: Mycket 1---2---3---4---5---6---7---8---9---10---11---12---13---14---15 Mycket

låg hög

varken låg

eller hög

# Hur fula/vackra uppfattar du att omgivningarna kring din färdväg är?

**Innerstaden**: Mycket 1---2---3---4---5---6---7---8---9---10---11---12---13---14---15 Mycket

fula vackra

varken fula

eller vackra

**Ytterstaden**: Mycket 1---2---3---4---5---6---7---8---9---10---11---12---13---14---15 Mycket

fula vackra

varken fula

eller vackra

# Hur mycket upplever du att din gångtur försvåras av färdvägens dragning?

# T.ex. dragning med många tvära svängar, omvägar, riktningsförändringar, sidbyten osv.

**Innerstaden**: Väldigt 1---2---3---4---5---6---7---8---9---10---11---12---13---14---15 Väldigt

lite mycket

varken lite

eller mycket

**Ytterstaden**: Väldigt 1---2---3---4---5---6---7---8---9---10---11---12---13---14---15 Väldigt

lite mycket

varken lite

eller mycket

# Hur mycket upplever du att din gångtur försvåras av färdvägens backighet? Utgå från färdvägen till och från arbets-/studieplatsen.

**Innerstaden**: Väldigt 1---2---3---4---5---6---7---8---9---10---11---12---13---14---15 Väldigt

lite mycket

varken lite

eller mycket

**Ytterstaden**: Väldigt 1---2---3---4---5---6---7---8---9---10---11---12---13---14---15 Väldigt

lite mycket

varken lite

eller mycket

# Hur mycket upplever du att din framkomlighet försämras av antalet rödljus

# under din färd till arbets-/studieplatsen?

**Innerstaden**: Väldigt 1---2---3---4---5---6---7---8---9---10---11---12---13---14---15 Väldigt

lite mycket

varken lite

eller mycket

**Ytterstaden**: Väldigt 1---2---3---4---5---6---7---8---9---10---11---12---13---14---15 Väldigt

lite mycket

varken lite

eller mycket

# Hur kort/lång upplever du att din färdväg är?

**Innerstaden**: Väldigt 1---2---3---4---5---6---7---8---9---10---11---12---13---14---15 Väldigt

kort lång

varken kort

eller lång

**Ytterstaden**: Väldigt 1---2---3---4---5---6---7---8---9---10---11---12---13---14---15 Väldigt

kort lång

varken kort

eller lång

1. **Om du har synpunkter på denna undersökning och dess frågor
   så skriv dem gärna här och vid behov fortsätt på baksidan.**
   ________________________________________________________________________________________________________________________________________________________________________________________________________________________________________________________________________________________________________________________________________________________________________________________________________________________________________________________________________________________________________________________________________________________________________________________________________________________________________________________________________________________________________________________________________________

**Stort tack för hjälpen!**
