# Supplementary material for: The heart rate method for estimating oxygen uptake: Analyses of reproducibility using a range of heart rates from cycle commuting
Source: PLoS One. 2019 Jul 24;14(7):e0219741. doi: 10.1371/journal.pone.0219741 (PMC6655643; doi:10.1371/journal.pone.0219741)
Supplement: S8 Methods — The original version in Swedish translated into English. (DOCX) [file pone.0219741.s008.docx]

**Questionnaire No 2**

**about physical activity**

**while commuting**

# Survey directed to you who walk or cycle to your place of work/study

#### Instructions for filling out this questionnaire

Put a cross in the box to answer, like this . If you mark a wrong box by mistake, then fill the whole box with colour, like this . Then put a cross in the correct box.

#### Questions about travelling time and relation to other modes of transport

1. **At what time have you usually walked/cycled from your home to your place of work/study during the last two weeks?**

Between 5.00 and 6.00 a.m.

Between 6.01 and 7.00 a.m.

Between 7.01 and 8.00 a.m.

Between 8.01 and 9.00 a.m.

Between 9.01 and 10.00 a.m.

Other time of the day ……….

1. **At what time have you usually walked/cycled from your place of work/study during the last two weeks?**

Between 2.00 and 3.00 p.m.

Between 3.01 and 4.00 p.m.

Between 4.01 and 5.00 p.m.

Between 5.01 and 6.00 p.m.

Between 6.01 and 7.00 p.m.

Between 7.01 and 8.00 p.m.

Other time of the day ……….

1. **How long time would your journey to your place of work/study take if, instead, you would choose** **the fastest route by public transport at the corresponding time of the day?**

**………** hours **………** minutes Don’t know

1. How long time would your journey to your place of work/study take if, instead, you would choose the fastest route by car at the corresponding time of the day?

**………** hours **………** minutes  Don’t know

**This is a question for you who walk:**

# What mode of transport would you choose if you could not walk all the way to your place of work/study?

Car
 Car, as passenger
 Public transport
 Moped
 Motor bike
 Bicycle
 Combination of means of conveyance. Which ones?……………………..

Other mode of conveyance: …………………….
 Don’t know

**This is a question for you who cycle:**

# What mode of transport would you choose if you could not cycle all the way to your place of work/study?

Car
 Car, as passenger
 Public transport
 Moped
 Motor bike
 Walking
 Combination of means of conveyance. Which ones?……………………..
 Other mode of conveyance: …………………….
 Don’t know

1. **Do you have a driving license for private car?**

Yes  No  Don’t know

1. **Do you usually have access to a private car?**

No

Yes, my own car
 Yes, a car in my household
 Yes, through a car pool
 Yes, it is possible for me to share a car journey with somebody else´s car
 Yes, several cars.
 Don’t know

1. **Do you have access to a car parking place at your place of work/study?**

No

Yes, free

Yes, subject to a fee

Don’t know

1. **What parking possibilities do you have near your home?**

I have my own parking place

Very good

Rather good

Rather poor

Very poor

Don’t know

1. **Is parking free/subject to a fee near your home?**

For example, if it is free during the night and on weekends while it is subject to a fee in the daytime, tick “subject to a fee”..

Free

Subject to a fee at certain times during the week.

Don’t know

1. **Have you had access to a travelcard for the local public transport during the last two weeks?**
   By travelcard we mean a card which is valid for a year, a season, a school term or a month.

No

Yes, my own

Yes, borrowed

Don’t know

## Background questions

1. **Where did you mainly live during your childhood and youth?**

Metropolitan area, including suburbs (e.g. Stockholm, Göteborg, Malmö)
 Other city/town
 Minor place
 Rural area
 Don’t know

1. **How did you in general get to school when you were around 12 years of age?**

School bus/school taxi most of the way

I was taken there by car

I cycled a short route (1 kilometre or less) to school

I cycled a long way (more than 1 km) to school

I walked a short way (1 kilometre or less) to school

I walked a long way (more than 1 kilometre) to school

Other mode of conveyance

Don’t remember

1. **Did you in your spare time engage in sport during your childhood and/or youth?**Mark one or more boxes!

No

Yes, in an athletics association and participated in competitions
 Yes, in an athletics association, but did not participate in competitions
 Yes, with friends and/or on my own
 Yes, with my family
 Don’t know

1. **Did you in your spare time engage in outdoor life during your childhood and/or youth?**Mark one or more boxes!

No

Yes, outdoor life in an association
 Yes, with friends and/or on my own
 Yes, with my family
 Don’t know

1. **Were you a member of an association dedicated to environmental issues during your childhood and/or youth?**

Yes  No  Don’t know

1. **What was your last mark at school in physical education?**

Mark: *……………*  Don’t know

## General questions

1. **What is your average income per month?**

By income we mean salary, wage, pension, renumeration, security, study allowances, income from self-employment.

No income at all

0-10 000 SEK

10 001 - 15 000 SEK

15 001 - 20 000 SEK

20 001 - 25 000 SEK

25 001 - 30 000 SEK

30 001 - 35 000 SEK

35 001 SEK or more

1. **What is your highest education level?**Tick one box only!

Elementary school (Folkskola)
 Compulsory school (nine years)
 Compulsory special school (for the intellectually disabled)
 Junior secondary school (Realskola)
 Vocational school or equivalent

Two-year continuation school (Fackskola)
 3-4 year upper secondary school
 Adult education centre (Folkhögskola)
 University or university college
 Other education, namely ………………..
 Don’t know

The following two questions are addressed to persons in work. If you are not working but studying etc, proceed directly to question No 23.

1. **Who is your main employer?**

Big private company

Small private company

Non-governmental organization/association
 Governmental agency or department
 Municipality
 County council

Don’t know

1. **In what professional branch are you occupied?**Choose the best-fitting option.

Agriculture, hunting and forestry

Manufacturing industry

Electricity, gas, heat and water supply

Building and construction

Wholesale and retail

Accommodation and catering services

Transport, storage

Postal and telecommunication services

Financial and insurance activities

Real estate and letting of immovable property

Business services, e.g. computer services

Research & development

Public administration and defence

Education

Health service, social service, veterinary service

Other public and personal services, e.g. culture, religion, recreation, sports.

Other, namely ……………….

1. **What kind of dwelling do you live in?**

Block of flats (rent)

Block of flats (cooperative)

Terraced house / detached house / single-family house (rent)

Terraced house / detached house / single-family house (own, cooperative)

Don’t know

1. **Do you share your dwelling with someone?**

I.e. with whom do you live during most of the week? You can tick several options!!

No

Yes, parents/siblings

Yes, spouse/cohab/partner

Yes, other adults

Yes, children, how many:……. 🡪 How old are they?

Indicate numbers at each relevant age group:

0-6 years …..

7-12 years …..

13-17 years …..

18 years or older .....

1. **Did you or your parents immigrate to Sweden?**

You may indicate more than one option

No

Yes, myself from………………………….…

Yes, my mother from……………………….…..

Yes, my father from ………………………………

Different option ……………………………

Don’t know

1. **Are you a member of a sports association?**

No

Yes, as leader

Yes, active, also in competitions

Yes, active in physical exercise

Yes, passive member

Don’t know

1. **Are you a member of an outdoor life association?**

No

Yes, active member

Yes, passive member

Don’t know

1. **Are you a member of an association dedicated to environmental issues?**

No

Yes, active member

Yes, passive member

Don’t know

#### Questions about your state of health and your lifestyle

1. **How is your general state of physical health?**

Very poor

Poor

Not very good

Acceptable

Rather good

Good

Very good

Don’t know

1. **How is your general state of mental health?**

Very poor

Poor

Not very good

Acceptable

Rather good

Good

Very good

Don’t know

1. **Have you been sick leave during the last 12 months?**

Do not include nursing of children.

No  Yes, in all around……… days  Don’t know

1. **Do you smoke?**

No

Yes, every day at least once

Yes, sometimes

Don’t know

1. **Do you have any medical/physical disability and/or asthma, allergic trouble preventing you from physical activity?**

No  Yes, namely……………………………  Don’t know

1. **Are you interested in participating in the third stage of this enquiry?**

    Yes  No  Don’t know

#### Question to you who have moved or changed place of work/study

1. If you have a different route than the one you indicated on the map in September 2004 and you still walk or cycle to your place of work/study, we ask you to indicate your new home address and the new address of your place of work/study.

New home address: New place of work/study:

# Street: …………………………. ……………………………

# Postal code: …………………….… ……………………………

**Place**: ……………………………..... ……………………………

Next, some questions will follow about how you perceive the environment in which you have walked or cycled on your way to your place of work/study during the last two weeks. Indicate your comprehensive impression during these weeks. We ask you to distinguish between experiences from the route passing through the inner city environment and through suburban environments, respectively (see Figure 1 below). Indicate experiences of the inner urban area on line 1 and of suburban areas on line 2, see the example in the box at the bottom of the page.

### Questions about your route

**The**

**northern suburban areas**

**Lill-Jans skogen**

**The inner urban area**

**The southern suburban areas**

**Brunnsviken**

**Östermalm**

**Kungsholmen**

**Södermalm**

**Årsta**

**Liljeholmen**

**Stora Essingen**

**Bromma**

**Vasastan**

**Solna**

**KTH**

**City**

##### Nacka

##### Gärdet

Figure 1. In this context, the inner urban area comprises the areas inside the dashed line and the suburban areas comprise the rest of Stockholm county. E.g. The Old Town = inner urban area, Täby and Huddinge = suburban areas.

**How to answer the questions.**

# Encircle the figure which corresponds best with your experience. If you make a mistake or change your mind, we ask you to put a cross over the wrong choice and a ring around the correct alternative. See the example.

**Inner urban:** Little 1---2---3---4---5---6---7---8---9---10---11---12---13---14---15 Much

Neither much

nor little

**Suburban:** Little 1---2---3---4---5---6---7---8---9---10---11---12---13---14---15 Much

Neither much

nor little

If you cycle/walk in both the environments, you shall mark both lines. If you first cycle in the southern suburban areas, then pass through the inner city and finish the journey in the northern suburbs, please indicate an average value for the two suburban parts of the journey.

### Questions about the environments in which you have cycled

All the questions below concern your comprehensive experience of your route as a cyclist to your place of work/study. Encircle the figure which corresponds best with your experience.

# How do you experience the environment on the whole along the route?

**Inner urban**: Very 1---2---3---4---5---6---7---8---9---10---11---12---13---14---15 Very

bad good

Neither bad

nor good

Suburban: Very 1---2---3---4---5---6---7---8---9---10---11---12---13---14---15 Very

bad good

Neither bad

nor good

1. **Do you think that, on the whole, the environment you cycle in stimulates/hinders**

**your commuting?**

**Inner urban**: Hinders 1---2---3---4---5---6---7---8---9---10---11---12---13---14---15 Stimulates

a lot a lot

Neither hinders

nor stimulates

**Suburban**: Hinders 1---2---3---4---5---6---7---8---9---10---11---12---13---14---15 Stimulates

a lot a lot

Neither hinders

nor stimulates

38. **How do you find the exhaust fume levels along your route?**

**Inner urban**: Very 1---2---3---4---5---6---7---8---9---10---11---12---13---14---15 Very

low high

Neither low

nor high

**Suburban**: Very 1---2---3---4---5---6---7---8---9---10---11---12---13---14---15 Very

low high

Neither low

nor high

**39. How do you find the noise levels along your route?**

**Inner urban**: Very 1---2---3---4---5---6---7---8---9---10---11---12---13---14---15 Very

low high

Neither low

nor high

**Suburban**: Very 1---2---3---4---5---6---7---8---9---10---11---12---13---14---15 Very

low high

Neither low

nor high

**40. How do you find the flow of motor vehicles (number of cars) along your route?**

**Inner urban**: Very 1---2---3---4---5---6---7---8---9---10---11---12---13---14---15 Very

low high

Neither low

nor high

**Suburban**: Very 1---2---3---4---5---6---7---8---9---10---11---12---13---14---15 Very

low high

Neither low

nor high

**41. How do you find the speeds of motor vehicles (taxis, lorries, ordinary cars, buses) along your route?**

**Inner urban**: Very 1---2---3---4---5---6---7---8---9---10---11---12---13---14---15 Very

low high

Neither low

nor high

**Suburban**: Very 1---2---3---4---5---6---7---8---9---10---11---12---13---14---15 Very

low high

Neither low

nor high

1. **How do you find other cyclists’ speeds along your route?**

**Inner urban**: Very 1---2---3---4---5---6---7---8---9---10---11---12---13---14---15 Very

low high

Neither low

nor high

**Suburban**: Very 1---2---3---4---5---6---7---8---9---10---11---12---13---14---15 Very

low high

Neither low

nor high

1. How do you as a cyclist find the congestion levels in mixed traffic, caused by all

types of vehicles, along your route?

**Inner urban**: Very 1---2---3---4---5---6---7---8---9---10---11---12---13---14---15 Very

low high

Neither low

nor high

**Suburban**: Very 1---2---3---4---5---6---7---8---9---10---11---12---13---14---15 Very

low high

Neither low

nor high

1. **How do you find the congestion levels caused by the number of cyclists on the**

**cycle paths/cycle lanes along your route?**

**Inner urban**: Very 1---2---3---4---5---6---7---8---9---10---11---12---13---14---15 Very

low high

Neither low

nor high

**Suburban**: Very 1---2---3---4---5---6---7---8---9---10---11---12---13---14---15 Very

low high

Neither low

nor high

1. **How do you find the occurrence of conflicts between you as a cyclist and other**

**road users (including pedestrians) along your route?**

**Inner urban**: Very 1---2---3---4---5---6---7---8---9---10---11---12---13---14---15 Very

low high

Neither low

nor high

**Suburban**: Very 1---2---3---4---5---6---7---8---9---10---11---12---13---14---15 Very

low high

Neither low

nor high

42. About how large part of your route consists of cycle paths/cycle lanes/cycle roads

separated from motor-car traffic? Encircle the approximate share.

**Inner urban**: 0 % ----10----20----30----40----50----60----70----80----90----100 %

**Suburban**: 0 % ----10----20----30----40----50----60----70----80----90----100 %

1. **How unsafe/safe do you feel in traffic as a cyclist along your route?**

**Inner urban**: Very 1---2---3---4---5---6---7---8---9---10---11---12---13---14---15 Very

unsafe safe

Neither unsafe

nor safe

**Suburban**: Very 1---2---3---4---5---6---7---8---9---10---11---12---13---14---15 Very

unsafe safe

Neither unsafe nor safe

1. **How do you find the availability of greenery (natural areas, parks, planted items, trees) along your route?**

**Inner urban**: Very 1---2---3---4---5---6---7---8---9---10---11---12---13---14---15 Very

low high

Neither low

nor high

**Suburban**: Very 1---2---3---4---5---6---7---8---9---10---11---12---13---14---15 Very

low high

Neither low

nor high

# How ugly/beautiful do you find the surroundings along your route?

**Inner urban**: Very 1---2---3---4---5---6---7---8---9---10---11---12---13---14---15 Very

ugly beautiful

Neither ugly

nor beautiful

**Suburban**: Very 1---2---3---4---5---6---7---8---9---10---11---12---13---14---15 Very

ugly beautiful

Neither ugly

nor beautiful

# To what extent do you feel that your cycle trip is made more difficult by the course of the route? For example a course with many sharp turns, detours, changes in directions, side changeovers etc.

**Inner urban**: Very 1---2---3---4---5---6---7---8---9---10---11---12---13---14---15 Very

little much

Neither little

nor much

**Suburban**: Very 1---2---3---4---5---6---7---8---9---10---11---12---13---14---15 Very

little much

Neither little

# nor much

# To what extent do you feel that your cycle trip is made more difficult by hilliness? Base this on the route to and from the place of work/study.

**Inner urban**: Very 1---2---3---4---5---6---7---8---9---10---11---12---13---14---15 Very

little much

Neither little

nor much

**Suburban**: Very 1---2---3---4---5---6---7---8---9---10---11---12---13---14---15 Very

little much

Neither little

# nor much

# To what extent do you feel that progress in traffic is worsened by the number

# of red lights during your trip to your place of work/study?

**Inner urban**: Very 1---2---3---4---5---6---7---8---9---10---11---12---13---14---15 Very

little much

Neither little

nor much

**Suburban**: Very 1---2---3---4---5---6---7---8---9---10---11---12---13---14---15 Very

little much

Neither little

# nor much

# How short/long do you experience your route to be?

**Inner urban**: Very 1---2---3---4---5---6---7---8---9---10---11---12---13---14---15 Very

short long

Neither short

nor long

**Suburban**: Very 1---2---3---4---5---6---7---8---9---10---11---12---13---14---15 Very

short long

Neither short

# nor long

All the following questions refer to your comprehensive experience as pedestrian of your route to your place of work/study. Circle the figure that matches your experience.

### Questions about the environments in which you have walked

# How do you experience the environment on the whole along the route?

**Inner urban**: Very 1---2---3---4---5---6---7---8---9---10---11---12---13---14---15 Very

bad good

Neither bad

nor good

Suburban: Very 1---2---3---4---5---6---7---8---9---10---11---12---13---14---15 Very

Bad good

Neither bad

nor good

1. **Do you think that, on the whole, the environment you walk in stimulates/hinders your commuting?**

**Inner urban**: Hinders 1---2---3---4---5---6---7---8---9---10---11---12---13---14---15 Stimulates

a lot a lot

Neither hinders

nor stimulates

**Suburban**: Hinders 1---2---3---4---5---6---7---8---9---10---11---12---13---14---15 Stimulates

a lot a lot

Neither hinders

nor stimulates

**51. How do you find the exhaust fume levels along your route?**

**Inner urban**: Very 1---2---3---4---5---6---7---8---9---10---11---12---13---14---15 Very

low high

Neither low

nor high

**Suburbs**: Very 1---2---3---4---5---6---7---8---9---10---11---12---13---14---15 Very

low high

Neither low

nor high

1. **How do you find the noise levels along your route?**

**Inner urban**: Very 1---2---3---4---5---6---7---8---9---10---11---12---13---14---15 Very

low high

Neither low

nor high

**Suburban**: Very 1---2---3---4---5---6---7---8---9---10---11---12---13---14---15 Very

low high

Neither low

nor high

1. **How do you find the flow of motor vehicles (number of cars) along your route?**

**Inner urban**: Very 1---2---3---4---5---6---7---8---9---10---11---12---13---14---15 Very

low high

Neither low

nor high

**Suburban**: Very 1---2---3---4---5---6---7---8---9---10---11---12---13---14---15 Very

low high

Neither low

nor high

1. **How do you find the speeds of motor vehicles (taxis, lorries, ordinary cars, buses)**

**along your route?**

**Inner urban**: Very 1---2---3---4---5---6---7---8---9---10---11---12---13---14---15 Very

low high

Neither low

nor high

**Suburban**: Very 1---2---3---4---5---6---7---8---9---10---11---12---13---14---15 Very

low high

Neither low

nor high

60. How do you find the congestion levels caused by the number of pedestrians along

your route?

**Inner urban**: Very 1---2---3---4---5---6---7---8---9---10---11---12---13---14---15 Very

low high

Neither low

nor high

**Suburban**: Very 1---2---3---4---5---6---7---8---9---10---11---12---13---14---15 Very

low high

Neither low

nor high

**61. How do you find the occurrence of conflicts between you as a pedestrian and other**

**road users (including pedestrians) along your route?**

**Inner urban**: Very 1---2---3---4---5---6---7---8---9---10---11---12---13---14---15 Very

low high

Neither low

nor high

**Suburban**: Very 1---2---3---4---5---6---7---8---9---10---11---12---13---14---15 Very

low high

Neither low

nor high

**62. How unsafe/safe do you feel in traffic as a pedestrian along your route?**

**Inner urban**: Very 1---2---3---4---5---6---7---8---9---10---11---12---13---14---15 Very

unsafe safe

Neither unsafe

nor safe

**Suburban**: Very 1---2---3---4---5---6---7---8---9---10---11---12---13---14---15 Very

unsafe safe

Neither unsafe

nor safe

**63. How do you find the availability of greenery (natural areas, parks, planted items,**

**trees) along your route?**

**Inner urban**: Very 1---2---3---4---5---6---7---8---9---10---11---12---13---14---15 Very

low high

Neither low

nor high

**Suburban**: Very 1---2---3---4---5---6---7---8---9---10---11---12---13---14---15 Very

low high

Neither low

nor high

# 64. How ugly/beautiful do you find the surroundings along your route?

**Inner urban**: Very 1---2---3---4---5---6---7---8---9---10---11---12---13---14---15 Very

ugly beautiful

Neither ugly

nor beautiful

**Suburban**: Very 1---2---3---4---5---6---7---8---9---10---11---12---13---14---15 Very

ugly beautiful

Neither ugly

nor beautiful

# 65. To what extent do you feel that your walking trip is made more difficult by the

# course of the route? For example a course with many sharp turns, detours,

# changes in directions, side changeovers etc.

**Inner urban**: Very 1---2---3---4---5---6---7---8---9---10---11---12---13---14---15 Very

little much

Neither little

nor much

**Suburban**: Very 1---2---3---4---5---6---7---8---9---10---11---12---13---14---15 Very

little much

Neither little

# nor much

# 66. To what extent do you feel that your walking trip is made more difficult by

# hilliness? Base this on the route to and from the place of work/study.

**Inner urban**: Very 1---2---3---4---5---6---7---8---9---10---11---12---13---14---15 Very

little much

Neither little

nor much

**Suburban**: Very 1---2---3---4---5---6---7---8---9---10---11---12---13---14---15 Very

little much

Neither little

# nor much

# 67. To what extent do you feel that progress in traffic is worsened by the number of red

# lights during your trip to your place of work/study?

**Inner urban**: Very 1---2---3---4---5---6---7---8---9---10---11---12---13---14---15 Very

little much

Neither little

nor much

**Suburban**: Very 1---2---3---4---5---6---7---8---9---10---11---12---13---14---15 Very

little much

Neither little

# nor much

# 68. How short/long do you experience your route to be?

**Inner urban**: Very 1---2---3---4---5---6---7---8---9---10---11---12---13---14---15 Very

short long

Neither short

nor long

**Suburban**: Very 1---2---3---4---5---6---7---8---9---10---11---12---13---14---15 Very

short long

Neither short

# nor long

**69. If you want to comment upon this survey and its questions you are welcome to write down your views here and, if you need, continue on the back page.** ________________________________________________________________________________________________________________________________________________________________________________________________________________________________________________________________________________________________________________________________________________________________________________________________________________________________________________________________________________________________________________________________________________________________________________________________________________________________________________________________________________________________________________________________

**Thanks a lot for your help!**
