# Supplementary material for: The heart rate method for estimating oxygen uptake: Analyses of reproducibility using a range of heart rates from cycle commuting
Source: PLoS One. 2019 Jul 24;14(7):e0219741. doi: 10.1371/journal.pone.0219741 (PMC6655643; doi:10.1371/journal.pone.0219741)
Supplement: S1 Results — (RTF) [file pone.0219741.s009.rtf]

S1 Results. The individual data, grouped by sex, that constitute the bases for Tables 6-11. 


Table 6. Reproducibility of HR-VO2 regression equations and correlation coefficients based on three submaximal work rates (model 1)(means ± SD).


Partici-pants	
HR-VO2 regression equations 
	
	Day 1	Day 2	
Males	y-intercept	slope	r	y-intercept	slope	r	
1     	-2.79	0.0465	0.999	-3.41	0.0539	0.998	
2     	-1.40	0.0318	0.985	-2.04	0.0362	1.000	
3    	-1.03	0.0277	0.997	-1.60	0.0326	0.985	
4   	-1.78	0.0382	0.998	-1.11	0.0308	0.998	
5   	-0.13	0.0153	0.989	-0.54	0.0202	0.992	
6     	-1.57	0.0279	1.000	-3.16	0.0393	1.000	
7   	-1.77	0.0304	0.999	-1.27	0.0274	0.999	
8      	-1.40	0.0304	1.000	-1.37	0.0301	1.000	
9   	-1.05	0.0236	0.999	-2.08	0.0316	0.999	
Mean	-1.44	0.0302	0.996	-1.84	0.0336	0.997	
SD	0.72	0.0087	0.005	0.95	0.0093	0.005	
							
Females							
1    	-0.70	0.0176	1.000	-0.63	0.0168	0.999	
2    	-0.87	0.0171	0.999	-1.41	0.0207	1.000	
3    	-0.86	0.0178	1.000	-1.17	0.0210	0.993	
4     	-0.92	0.0185	0.985	-1.59	0.0229	1.000	
5     	-1.49	0.0238	0.999	-1.42	0.0235	1.000	
6    	-2.20	0.0296	0.999	-1.59	0.0263	0.996	
7    	-1.15	0.0195	0.998	-1.31	0.0198	1.000	
8   	-0.95	0.0212	0.995	-1.38	0.0260	0.992	
9    	-1.50	0.0210	0.990	-0.88	0.0178	1.000	
10   	-1.75	0.0259	1.000	-2.07	0.0286	0.997	
Mean	-1.24	0.0212	0.996	-1.35	0.0223	0.998	
SD	0.48	0.0041	0.005	0.40	0.0038	0.003	


Table 7. The estimated levels of VO2 based on the HR-VO2 regression equations in day 1 and 2 (model 1) and three levels of HR from cycle commuting (means ± SD, and coefficients of variation (CV)).


Partici-pants	
HR at field and estimations of VO2 based on three levels of HR  
and the HR-VO2 regression equations at day 1 and 2 
	
	Lowest fifth of HR	Middle fifth of HR	Highest fifth of HR	
Males	HR
F1	VO2:1	VO2:2	Abs diff	% diff	HR F3	VO2:   1	VO2:2	Abs diff	% diff	HR F5	VO2:1	VO2:2	Abs diff	     %
diff	
1     	123	2.92	3.22	0.29	10.0	155	4.40	4.93	0.53	12.0	163	4.78	5.37	0.59	12.3	
2     	111	 2.13	1.98	-0.15	-6.94	135	2.88	2.84	-0.04	-1.49	149	3.35	3.37	0.02	0.64	
3    	125	 2.42	 2.47	0.04	 1.85	 144	   2.95	 3.09	 0.14	 4.68	 158	 3.33	 3.54	   0.21	    6.18	
4   	102	2.12	2.03	-0.09	-4.11	131	3.23	2.92	-0.30	-9.35	150	3.96	3.52	-0.44	-11.2	
5    	95	1.32	1.39	0.06	4.62	116	1.64	1.81	0.16	9.95	129	1.85	2.08	0.23	12.4	
6     	111	1.52	1.20	-0.32	-21.3	130	2.07	1.96	-0.10	-4.95	144	2.43	2.48	0.05	1.94	
7   	115	1.72	1.87	0.15	8.99	131	2.21	2.32	0.11	4.76	144	2.60	2.67	0.07	2.57	
8      	125	2.40	2.39	0.00	-0.14	144	2.97	2.97	-0.01	-0.30	153	3.25	3.24	-0.01	-0.36	
9   	115	1.67	1.55	-0.11	-6.77	138	2.21	2.28	0.07	3.22	147	2.42	2.56	0.14	5.91	
Mean	113	2.02	2.01	-0.01	-1.53	136	2.73	2.79	0.06	2.06	149	3.11	3.20	0.09	3.37	
SD	10.2	0.51	0.62	0.18	9.67	11.0	0.82	  0.93	0.23	6.84	9.53	0.90	0.96	0.27	7.18	
CV				6.33					5.81					6.07		
Females																
1    	99	1.04	1.03	-0.01	-0.49	129	1.57	1.54	-0.03	-1.88	149	1.92	1.87	-0.05	-2.36	
2    	124	1.25	1.15	-0.10	-7.91	145	1.61	1.59	-0.02	-1.44	161	1.88	1.91	0.03	1.78	
3    	128	1.42	1.52	0.10	6.75	143	1.69	1.83	0.14	8.53	161	2.02	2.22	0.20	10.1	
4     	125	1.39	1.27	-0.12	-8.65	141	1.70	1.65	-0.05	-2.85	153	1.91	1.92	0.00	0.19	
5     	129	1.59	1.63	0.04	2.47	141	1.86	1.90	0.04	1.91	154	2.18	2.21	0.03	1.46	
6    	119	1.33	1.55	0.21	15.9	136	1.83	1.98	0.16	8.56	144	2.08	2.21	0.13	6.19	
7 	117	1.14	1.02	-0.12	-10.5	146	1.69	1.58	-0.11	-6.54	161	1.99	1.88	-0.11	-5.32	
8  	106	1.29	1.37	0.08	6.17	130	1.80	2.00	0.20	10.9	144	2.10	2.37	0.26	12.6	
9    	114	0.89	1.14	0.25	28.7	140	1.43	1.60	0.17	12.0	150	1.65	1.79	0.14	8.45	
10   	119	1.33	1.33	0.00	0.09	139	1.85	1.90	0.06	3.00	157	2.31	2.42	0.10	4.49	
Mean	118	1.27	1.30	0.03	3.25	139	1.70	1.76	0.05	3.22	154	2.00	2.08	0.08	3.75	
SD	9.76	0.20	0.22	0.13	12.0	5.67	0.14	0.18	0.11	6.45	6.55	0.18	0.23	0.11	5.65	
CV				7.20					4.38					3.93		


Table 8. Reproducibility of HR-VO2 regression equations and correlation coefficients based on three submaximal and a maximal work rate (model 2)(means ± SD).


Partici-pants	
HR-VO2 regression equations 
	
	Day 1	Day 2	
Males	y-intercept	slope	r	y-intercept	slope	r	
1	-3.60	0.0543	0.998	-4.00	0.0597	0.999	
2	-1.50	0.0327	0.998	-1.69	0.0332	0.999	
3	-1.28	0.0300	0.999	-1.55	0.0322	0.998	
4	-1.26	0.0330	0.996	-1.13	0.0310	0.999	
5	-1.01	0.0223	0.943	-1.00	0.0242	0.991	
6	-2.40	0.0346	0.992	-3.28	0.0402	1.000	
7	-1.89	0.0315	0.999	-1.12	0.0261	0.999	
8	-1.61	0.0324	0.999	-1.34	0.0298	1.000	
9	-0.73	0.0210	0.993	-1.84	0.0297	0.998	
Mean	-1.70	0.0324	0.991	-1.88	0.0340	0.998	
SD	0.86	0.0095	0.018	1.05	0.0106	0.003	
							
Females							
1	-0.84	0.0190	0.997	-0.71	0.0176	0.999	
2	-1.26	0.0203	0.992	-1.85	0.0242	0.995	
3	-1.06	0.0195	0.996	-1.26	0.0217	0.997	
4	-0.82	0.0176	0.991	-1.59	0.0229	1.000	
5	-1.30	0.0222	0.998	-1.21	0.0217	0.997	
6	-1.63	0.0247	0.991	-0.67	0.0180	0.960	
7	-1.21	0.0199	0.999	-1.43	0.0208	0.999	
8	-0.60	0.0177	0.992	-0.90	0.0213	0.987	
9	-1.45	0.0207	0.997	-1.00	0.0188	0.999	
10	-1.82	0.0265	1.000	-1.79	0.0260	0.998	
Mean	-1.20	0.0208	0.995	-1.24	0.0213	0.993	
SD	0.38	0.0029	0.004	0.42	0.0027	0.012	


Table 9. The estimated levels of VO2 based on the HR-VO2 regression equations in day 1 and 2 (model 2) and three levels of HR from cycle commuting (means ± SD, and coefficients of variation (CV)).


Partici-pant	
HR at field and estimations of VO2 based on three levels of HR
and the HR-VO2 regression equations at day 1 and 2
 	
	Lowest fifth of HR	Middle fifth of HR	Highest fifth of HR	
Males	HR
F1	VO2:1	VO2:2	Abs diff	% diff	HR F3	VO2:   1	VO2:2	Abs diff	% diff	HR F5	VO2:1	VO2:2	Abs diff	     %
diff	
1     	123	3.08	3.34	0.26	8.54	155	4.81	5.24	0.43	9.04	163	5.25	5.73	0.48	9.12	
2     	111	2.13	1.99	-0.14	-6.62	135	2.90	2.77	-0.13	-4.44	149	3.38	3.26	-0.12	-3.60	
3    	125	2.47	2.46	0.00	-0.11	144	3.04	3.08	0.04	1.29	158	3.45	3.52	0.07	2.02	
4   	102	2.10	2.03	-0.07	-3.51	131	3.06	2.93	-0.13	-4.31	150	3.69	3.52	-0.17	-4.61	
5   	95	1.11	1.30	0.19	17.2	116	1.57	1.80	0.23	14.7	129	1.87	2.13	0.26	13.67	
6   	111	1.44	1.19	-0.26	-17.9	130	2.12	1.97	-0.15	-7.05	144	2.57	2.49	-0.08	-2.95	
7   	115	1.72	1.87	0.15	8.83	131	2.23	2.30	0.06	2.85	144	2.64	2.63	-0.01	-0.21	
8      	125	2.44	2.38	-0.06	-2.33	144	3.06	2.95	-0.11	-3.48	153	3.35	3.22	-0.13	-3.88	
9   	115	1.68	1.57	-0.11	-6.53	138	2.16	2.25	0.09	4.17	147	2.35	2.52	0.17	7.17	
Mean	113	2.02	2.01	0.00	-0.27	136	2.77	2.81	0.04	1.41	149	3.17	3.23	0.05	1.86	
	SD	10.2	0.60	0.66	0.17	10.4	11.0	0.93	1.02	0.20	7.12	9.53	0.98	1.06	0.22	6.6	
CV				6.02					4.99					4.75		
Females																
1    	99	1.03	1.03	-0.01	-0.47	129	1.61	1.57	-0.05	-2.96	149	1.99	1.91	-0.08	-3.79	
2    	124	1.26	1.15	-0.11	-9.03	145	1.69	1.66	-0.03	-1.88	161	2.01	2.04	0.03	1.48	
3    	128	1.44	1.52	0.08	5.46	143	1.73	1.84	0.11	6.44	161	2.09	2.24	0.15	7.27	
4     	125	1.38	1.27	-0.11	-8.24	141	1.67	1.64	-0.03	-1.61	153	1.88	1.91	0.04	1.90	
5     	129	1.57	1.60	0.03	1.83	141	1.83	1.85	0.02	1.26	154	2.12	2.14	0.02	0.77	
6    	119	1.32	1.48	0.16	12.4	136	1.73	1.78	0.05	2.97	144	1.94	1.93	0.00	-0.26	
7    	117	1.13	1.01	-0.12	-10.5	146	1.69	1.60	-0.09	-5.47	161	2.00	1.92	-0.08	-3.94	
8   	106	1.27	1.36	0.09	7.07	130	1.70	1.87	0.18	10.4	144	1.95	2.18	0.23	11.7	
9  	114	0.90	1.14	0.24	26.3	140	1.43	1.62	0.19	13.0	150	1.65	1.81	0.17	10.2	
10   	119	1.33	1.31	-0.02	-1.74	139	1.86	1.83	-0.03	-1.78	157	2.34	2.30	-0.04	-1.80	
Mean	118	1.26	1.28	0.02	2.31	139	1.69	1.73	0.03	2.04	154	2.00	2.04	0.04	2.35	
SD	9.76	0.20	0.21	0.12	11.2	5.67	0.12	0.12	0.10	6.12	6.55	0.18	0.17	0.11	5.55	
CV				6.74					 4.03					3.71		


Table 10. Differences between model 1 and 2 in HR-VO2 regression equations and correlation coefficients (means ± SD).


Partici-pants	
Differences between model 1 and 2 in the constituents of HR-VO2   regression equation and correlation coefficient in day 1 and 2
	
	Day 1	Day 2	
Males	y-intercept	slope	r	y-intercept	slope	r	
1     	-0.80	0.0078	-0.002	-0.59	0.0058	0.001	
2     	-0.10	0.0009	0.013	0.34	-0.0030	-0.001	
3    	-0.24	0.0023	0.002	0.05	-0.0004	0.014	
4   	0.52	0.0052	-0.002	-0.02	0.0002	0.002	
5    	-1.14	0.0070	-0.046	-0.47	0.0040	-0.001	
6     	-0.82	0.0067	-0.008	-0.11	0.0009	0.000	
7   	-0.12	0.0011	0.001	0.15	-0.0013	0.000	
8      	-0.20	0.0020	-0.001	0.03	-0.0003	0.000	
9   	0.31	-0.0026	-0.006	0.24	-0.0019	-0.001	
Mean	-0.29	0.0022	-0.005	-0.04	0.0004	0.002	
SD	0.54	0.0044	0.016	0.31	0.0028	0.005	
							
Females							
1    	-0.14	0.0014	-0.003	-0.08	0.0008	-0.001	
2    	-0.39	0.0032	-0.008	-0.44	0.0035	-0.005	
3    	-0.20	0.0017	-0.003	-0.09	0.0007	0.004	
4     	0.10	-0.0009	0.006	0.00	0.0000	0.000	
5     	0.19	-0.0016	-0.002	0.21	-0.0018	-0.003	
6    	0.57	-0.0049	-0.009	0.93	-0.0083	-0.036	
7    	-0.06	0.0004	0.001	-0.12	0.0010	0.000	
8   	0.35	-0.0035	-0.003	0.49	-0.0047	-0.005	
9    	0.04	-0.0003	0.007	-0.12	0.0010	-0.001	
10   	-0.07	0.0006	0.000	0.29	-0.0026	0.001	
Mean	0.04	-0.0004	-0.001	0.10	-0.0010	-0.004	
SD	0.28	0.0024	0.005	0.39	0.0034	0.011	


Table 11. Differences between model 1 and 2 in the estimated VO2 based on three levels of HR as well as in absolute and relative differences (means ± SD).


Partici-pants	
Differences between model 1 and 2 in estimations of VO2 based on 
the HR-VO2 regression equations on day 1 and 2
	
	Lowest fifth of HR	Middle fifth of HR	Highest fifth of HR	
Males	VO2
:1	VO2
:2	Abs diff	% diff	VO2
:1	VO2
:2	Abs diff	% diff	VO2
:1	VO2:2	Abs diff	     %
diff	
1     	0.16	0.13	-0.03	-1.49	0.40	0.31	-0.09	-2.96	0.47	0.36	-0.11	-3.20	
2     	0.00	0.01	0.01	0.32	0.02	-0.06	-0.09	-2.95	0.04	-0.11	-0.14	-4.23	
3    	0.04	0.00	-0.05	-1.96	0.09	-0.01	-0.10	-3.39	0.12	-0.02	-0.14	-4.16	
4   	-0.02	0.00	0.01	0.59	-0.17	0.00	0.17	5.04	-0.27	0.01	0.27	6.60	
5    	-0.22	-0.09	0.13	12.6	-0.07	0.00	0.07	4.70	0.02	0.05	0.03	1.28	
6     	-0.08	-0.01	0.07	3.39	0.05	0.00	-0.05	-2.10	0.14	0.02	-0.12	-4.89	
7   	0.00	0.00	0.00	-0.15	0.02	-0.02	-0.04	-1.91	0.03	-0.04	-0.07	-2.78	
8      	0.05	-0.01	-0.05	-2.20	0.08	-0.01	-0.10	-3.18	0.10	-0.02	-0.12	-3.52	
9   	0.01	0.02	0.00	0.24	-0.05	-0.03	0.02	0.95	-0.07	-0.04	0.03	1.25	
Mean	-0.01	0.00	0.01	1.26	0.04	0.02	-0.02	-0.64	0.07	0.02	-0.04	-1.52	
SD	0.10	0.05	0.06	4.58	0.16	0.11	0.09	3.39	0.19	0.13	0.14	3.80	
													
Females													
1    	0.00	0.00	0.00	0.01	0.04	0.02	-0.02	-1.08	0.07	0.04	-0.03	-1.43	
2    	0.01	-0.01	-0.01	-1.12	0.08	0.07	-0.01	-0.44	0.13	0.12	0.00	-0.30	
3    	0.02	0.00	-0.02	-1.30	0.05	0.01	-0.03	-2.09	0.08	0.03	-0.05	-2.80	
4     	-0.01	0.00	0.01	0.41	-0.03	0.00	0.02	1.24	-0.04	0.00	0.03	1.71	
5     	-0.02	-0.03	-0.01	-0.64	-0.03	-0.05	-0.01	-0.66	-0.06	-0.07	-0.02	-0.69	
6    	-0.02	-0.06	-0.05	-3.46	-0.10	-0.20	-0.11	-5.58	-0.14	-0.27	-0.13	-6.45	
7    	-0.01	-0.01	0.00	0.00	0.00	0.02	0.02	1.07	0.01	0.04	0.03	1.38	
8   	-0.02	-0.01	0.01	0.90	-0.10	-0.12	-0.02	-0.44	-0.15	-0.19	-0.04	-0.85	
9    	0.01	-0.01	-0.02	-2.45	0.00	0.02	0.01	1.00	0.00	0.03	0.03	1.70	
10   	0.00	-0.02	-0.02	-1.83	0.01	-0.08	-0.09	-4.78	0.02	-0.12	-0.15	-6.30	
Mean	0.00	-0.01	-0.01	-0.95	-0.01	-0.03	-0.02	-1.18	-0.01	-0.04	-0.03	-1.40	
SD	0.01	0.02	0.02	1.36	0.06	0.08	0.04	2.36	0.09	0.12	0.06	2.99	
